# High-dimensional entanglement witnessed by correlations in arbitrary bases

**DOI:** 10.1038/s41534-025-00990-6

**Published:** 2025-03-19

**Authors:** Nicky Kai Hong Li, Marcus Huber, Nicolai Friis

**Affiliations:** 1 Atominstitut, Technische Universität Wien, Stadionallee 2, 1020 Vienna, Austria; 2 Vienna Center for Quantum Science and Technology, TU Wien, 1020 Vienna, Austria; 3 Institute for Quantum Optics and Quantum Information (IQOQI), Austrian Academy of Sciences, Boltzmanngasse 3, 1090 Vienna, Austria

**Keywords:** Quantum information, Theoretical physics

## Abstract

Certifying entanglement is an important step in the development of many quantum technologies, especially for higher-dimensional systems, where entanglement promises increased capabilities for quantum communication and computation. A key feature distinguishing entanglement from classical correlations is the occurrence of correlations for complementary measurement bases. In particular, mutually unbiased bases (MUBs) are a paradigmatic example that is well-understood and routinely employed for entanglement certification. However, implementing unbiased measurements exactly is challenging and not generically possible for all physical platforms. Here, we extend the entanglement-certification toolbox from correlations in MUBs to arbitrary bases. This practically significant simplification paves the way for efficient characterizations of high-dimensional entanglement in a wide range of physical systems. Furthermore, we introduce a simple three-MUBs construction for all dimensions without using the Wootters–Fields construction, potentially simplifying experimental requirements when measurements in more than two MUBs are needed, especially in high-dimensional settings.

## Introduction

Entanglement is an important signature of “quantumness” and a central resource in quantum information processing. In particular, it is a crucial ingredient to achieving quantum advantages in many communication^1–5^, metrological^6,7^, and computational tasks^8^. Consequently, continuous efforts are being made to develop mathematical tools for the detection and quantification of entanglement in experiments^9^.

The certification of high-dimensional entanglement is of particular relevance to setups that use multilevel quantum systems to store and process information^10,11^. Entanglement in higher dimensions can be more robust to noise and can allow for higher data throughput when used for teleportation, such that the performance of many quantum information-processing tasks improves with the dimension of the accessible entanglement resources. For example, using higher-dimensional entanglement can improve secure key rates in quantum key distribution^12^ and benefit a wide range of quantum technologies such as entanglement-enhanced imaging^13–15^.

Moreover, entanglement certification can serve as a benchmark for quantum computers and simulators: If a device is supposed to output states with high-dimensional entanglement, then certifying the latter in the actual outputs can indicate how well the device is functioning^11,16^. At the same time, entanglement certification can increase one’s confidence that a quantum advantage can be achieved since the hardness of classically simulating a many-body system increases with the amount of entanglement^17–19^.

A central intuition behind entanglement detection is that entanglement leads to correlations between outcomes of local measurements in two or more complementary bases. In this context, complementarity is typically approached via the extremal case of mutually unbiased bases (MUBs). For a quantum system prepared in any basis state of any one of these bases, all measurement outcomes for any of the other MUBs are equally likely, i.e., knowing the measurement outcome in one basis tells us nothing about the outcomes in the complementary bases. Consequently, MUBs have been at the centre of many existing entanglement-detection methods^10,20–23^. Correlations measured in MUBs can in turn be used to bound well-defined figures of merit that quantify how strongly entangled the underlying state is. An example of such a quantity is the Schmidt number^24^—a generalization of the Schmidt rank for mixed states—that is used here to quantify entanglement dimensionality.

However, while some systems allow one to freely select the measurement bases (e.g., via spatial light modulators and single-mode fibres for spatial degrees-of-freedom of photons^10,22^), this is generically not the case for all setups. The inability to measure in the desired MUBs often goes hand in hand with (but does not mathematically imply) the impossibility of carrying out tomographically complete sets of measurements. For previous approaches to certifying high-dimensional entanglement, such limited control has been prohibitively restrictive.

Nevertheless, classical correlations cannot simultaneously be arbitrarily strong for any set of bases that are complementary in the sense of corresponding to non-commuting observables. Indeed, the detection of bipartite entanglement from measurements in arbitrary bases can, in principle, be achieved via entropic uncertainty relations^25,26^, which provide a lower bound on the entanglement cost^27^, but not on the Schmidt number.

In this work, we fill this gap by proposing a family of Schmidt-number witnesses based on correlations in at least two coordinated local orthonormal bases which can be chosen arbitrarily. We provide analytic upper bounds of the corresponding witness operators evaluated on any bipartite state with Schmidt number at most *k* (Theorem 1 & Lemma 1). The main advantage of our method is that the upper bounds depend only on the absolute values of the basis-vector overlaps and are independent of the relative phases between the measurement bases, which are often not directly measurable in experiments. Therefore, our witness significantly simplifies the requirements for certifying high-dimensional entanglement in experiments across a wider range of platforms. We also show that the bounds are the tightest when the underlying measurement bases are mutually unbiased (Corollary 1), confirming the intuition that MUBs are optimal for entanglement detection within this framework.

In addition, we analytically lower bound the *entanglement fidelity* (or the *singlet fraction*^28,29^)—the maximum fidelity of a bipartite state with any two-qudit maximally entangled state—using our witness (Theorem 1 & Lemma 1). This provides an alternative way to quantify high-dimensional entanglement. Next, we demonstrate the effectiveness of our Schmidt-number witnesses with two examples: the two-qudit isotropic state and the noisy two-qudit purified thermal states, and evaluate the difference between the actual entanglement fidelity and our bound for these examples (see also the Supplementary Information which includes the detailed analyses of the examples of isotropic states and noisy purified thermal states, discussions regarding the applications of random measurement bases and AMUBs, the proofs of eqs. (12) and (13) and Lemma 2, and refs. ^30–47^). To complete our analysis, we compare the white-noise tolerances of our witnesses with those proposed in ref. ^10^. We also discuss the possibility of using random measurement bases in high dimensions or approximately MUBs (AMUBs) (see the Supplementary Information) to witness Schmidt numbers and propose a simple (and, to the best of our knowledge, new) construction of three MUBs for all dimensions without using the Wootters-Fields construction^48^. Finally, we compare various existing methods for certifying high-dimensional entanglement with our method in Table 1.

**Table 1:** A comparison of different methods for the verification of high-dimensional entanglement

Methods	Features and experimental/computational requirements
Our witness	• Measure in at least two coordinated local (arbitrary) orthonormal bases • Only need to know the (minimum and maximum) absolute values of the overlaps between the local measurement bases
Reference ^10^’s witness	• Precise control over the absolute values & the complex phases of the overlaps between different local measurement bases and within each basis • Measure in the computational basis + at least one coordinated “tilted” basis
SDP witness^55^	• Treat bases bias as imperfect implementations of measurements in MUBs • Memory issue/long computational runtime for large dimensions • Efficient only for small dimensions in which case it is possible to obtain tighter bounds compared to our witness
Entropic uncertainty relationship^25,27^ [eq. (17.135) in ref. ^56^]	• Need to know the absolute values of the bases overlaps of only one party and the classical entropies corresponding to the two parties’ measurements • Can lower bound the distillable entanglement instead of the Schmidt number
Generalized Bell inequalities^57,58^	• To witness Schmidt number without any measurement assumptions, require non-trivial optimization over all possible local measurements and all states with Schmidt number ≤ *k* for every local dimension, which is computationally costly • Certify lower Schmidt numbers than other methods can in general • Standard approaches involve finding the largest eigenvalue corresponding to eigenvectors with Schmidt rank ≤ *k* of the Bell operator^59^ associated with restricted measurement settings^58,60^ to keep optimization problems numerically tractable (more specifically, ref. ^58^ uses physical arguments to restrict the maximization of the Bell operator’s expectation value to restricted sets of states with different maximum Schmidt numbers—referred to therein as entanglement dimensions—of which the union is believed to contain the experimental states) → also require measurement assumptions and are computationally feasible only for small dimensions
Correlation-matrix norms from randomized measurements^61^	• Independent of the relative reference frame between the two parties as the matrix $p$-norms for all even $p\in {\mathbb{N}}$ of the correlation matrix remain unchanged under any local unitary transformation of a bipartite state^61^. • Require sampling local unitaries randomly from (Haar measure or) *t*-designs, where exact sampling is highly inefficient^62^. • While approximate sampling from ${\mathrm{t}}$-designs can be efficient^63^, it is unclear how approximate sampling can affect the Schmidt -number witness in ref. ^61^. • Analytic bounds of the 2- and 4-norms of the correlation matrices corresponding to states with Schmidt number ≤ *k* are known only for *k* = 2 • For certifying Schmidt number ≥ 3, require numerical optimizations which can be computationally costly for large dimensions and the bounds can be loose since there are no tighter known constraints on the singular values of the correlation matrix other than the purity bound $\text{Tr}(\rho^2)<1$ (see Appendix C of ref. ^61^.)

## Results

### Background and notation

To detect bipartite entanglement, parties A and B measure their shared state *ρ*_*A* *B*_ in *m* local bases with global projectors $| {e}_{a}^{z}\left.\right\rangle \left\langle \right.{e}_{a}^{z}| \otimes | {\tilde{e}}_{a}^{z*} \left.\right\rangle \left\langle \right.{\tilde{e}}_{a}^{z*} |$ where ${\left\{| {e}_{a}^{z}\left.\right\rangle \right\}}_{a = 0}^{d-1}$ is the *z*-th orthonormal basis of the *m* bases, $| {\phi }^{* }\left.\right\rangle$ denotes the complex conjugate of the state $| \phi \left.\right\rangle$ with respect to the computational basis ${\left\{| i\left.\right\rangle \right\}}_{i = 0}^{d-1}$, and $| {\tilde{e}}_{a}^{z*} \left.\right\rangle := U| {e}_{a}^{z*} \left.\right\rangle$ with *U*  ∈  *U*(*d*) fixed for all *a* and *z*. Note that we do not require the *m* measurement bases to be MUBs as in ref. ^23^. The entanglement witness is then defined to be the sum of the probabilities of all matching outcomes in all matching pairs of bases, i.e.,

1 $${{\mathcal{S}}}_{d}^{(m)}({\rho }_{AB})=\mathop{\sum }\limits_{z=1}^{m}\mathop{\sum }\limits_{a=0}^{d-1}\left\langle \right.{e}_{a}^{z},{\tilde{e}}_{a}^{z*} | {\rho }_{AB}| {e}_{a}^{z},{\tilde{e}}_{a}^{z*} \left.\right\rangle .$$

In Theorem 1, we show how the upper bound of eq. (1) depends on the Schmidt number *k*(*ρ*_*A* *B*_)^24^ of the state *ρ*_*A* *B*_, which is defined as

2 $$k({\rho }_{AB}):= \mathop{\inf }\limits_{{\mathcal{D}}({\rho }_{AB})}\left\{\mathop{\max }\limits_{{\{({p}_{i},| {\psi }_{i}\left.\right\rangle )\}}_{i}}\,\text{rank}({\text{Tr}}_{B}| {\psi }_{i}\left.\right\rangle \left\langle \right.{\psi }_{i}| )\right\},$$

where ${\mathcal{D}}(\rho )$ is the set of all pure-state decompositions, ${\{({p}_{i},| {\psi }_{i}\left.\right\rangle )\}}_{i}$, of $\rho ={\sum }_{i}{p}_{i}| {\psi }_{i}\left.\right\rangle \left\langle \right.{\psi }_{i}|$ and ${\{{p}_{i}\}}_{i}$ is a probability distribution. In addition, we show that the maximum fidelity of *ρ*_*A* *B*_ with any maximally entangled state,

3 $${\mathcal{F}}({\rho }_{AB}):= \mathop{\max }\limits_{{U}_{A}}\left\langle \right.{\Phi }_{d}^{+}| ({U}_{A}\otimes {{\mathbb{1}}}_{B}){\rho }_{AB}{({U}_{A}\otimes {{\mathbb{1}}}_{B})}^{\dagger }| {\Phi }_{d}^{+}\left.\right\rangle ,$$

where the maximization is over all unitaries *U*_*A*_ acting on subsystem *A* and $| {\Phi }_{d}^{+}\left.\right\rangle =\frac{1}{\sqrt{d}}\mathop{\sum }\nolimits_{i = 0}^{d-1}| ii\left.\right\rangle$, can be lower bounded using the quantity ${{\mathcal{S}}}_{d}^{(m)}({\rho }_{AB})$. From now on, we call ${\mathcal{F}}({\rho }_{AB})$ the *entanglement fidelity* (also known as the *singlet fraction* in the case of qubits^28,29^) of $\rho_{AB}$.

Let us define the maximum and minimum overlaps between two bases *z* and ${z}^{{\prime} }$ as ${c}_{\max }^{z,{z}^{{\prime} }}=\mathop{\max }\nolimits_{a,{a}^{{\prime} }}| \left\langle \right.{e}_{a}^{z}| {e}_{{a}^{{\prime} }}^{{z}^{{\prime} }}\left.\right\rangle {| }^{2}$ and ${c}_{\min }^{z,{z}^{{\prime} }}=\mathop{\min }\nolimits_{a,{a}^{{\prime} }}| \left\langle \right.{e}_{a}^{z}| {e}_{{a}^{{\prime} }}^{{z}^{{\prime} }}\left.\right\rangle {| }^{2}$, respectively. We then define ${\mathcal{C}}={\{| \left\langle \right.{e}_{a}^{z}| {e}_{{a}^{{\prime} }}^{{z}^{{\prime} }}\left.\right\rangle {| }^{2}\}}_{a,{a}^{{\prime} },z\ne {z}^{{\prime} }}$ ($\overline{{\mathcal{C}}}={\{({c}_{\max }^{z,{z}^{{\prime} }},{c}_{\min }^{z,{z}^{{\prime} }})\}}_{z\ne {z}^{{\prime} }}$) to be the set that contains all (pairs of maximum and minimum) overlaps between any two different measurement bases.

### Schmidt-number witness & entanglement-fidelity bound

We now present our main results that use the expectation value ${{\mathcal{S}}}_{d}^{(m)}$ to infer lower bounds on the Schmidt number and entanglement fidelity of the state *ρ*_*A* *B*_.

#### Theorem 1

For any bipartite state *ρ*_*A* *B*_ of equal local dimension *d* and Schmidt number at most *k*, it holds that

4 $${{\mathcal{S}}}_{d}^{(m)}({\rho }_{AB})\le \frac{k(m-{\mathcal{T}}({\mathcal{C}}))}{d}+{\mathcal{T}}({\mathcal{C}})=:{{\mathcal{B}}}_{k},$$

where the upper bound ${{\mathcal{B}}}_{k}$ depends on the integers *d* and *k*, the number of measurement bases *m*, and a quantity ${\mathcal{T}}({\mathcal{C}})$ which depends on the set of bases overlaps. More specifically, ${\mathcal{T}}({\mathcal{C}}):= \min \{\lambda ({\mathcal{C}}),m\}$, $\lambda ({\mathcal{C}}):= \frac{1}{2}\left(1+\sqrt{1+2d{\sum }_{z\ne {z}^{{\prime} }}{G}^{z,{z}^{{\prime} }}}\right)\ge 1$, and ${G}^{z,{z}^{{\prime} }}:= 1-(d+1){c}_{\min }^{z,{z}^{{\prime} }}+\frac{1}{d}{\sum }_{a,{a}^{{\prime} }}| \left\langle \right.{e}_{a}^{z}| {e}_{{a}^{{\prime} }}^{{z}^{{\prime} }}\left.\right\rangle {| }^{4}$. Furthermore, the entanglement fidelity of *ρ*_*AB*_ can be lower bounded as follows:

5 $${\mathcal{F}}({\rho }_{AB})\ge \max \left\{0,\frac{{\mathcal{S}}_{d}^{{(m)}}({\rho }_{AB})-{{\mathcal{T}}}({{\mathcal{C}}})}{m-{{\mathcal{T}}}({{\mathcal{C}}})}\right\}=:{{\mathcal{F}}}_{m}.$$

The proof of Theorem 1 is given in full in the Methods. Theorem 1 implies that if the measured quantity ${{\mathcal{S}}}_{d}^{(m)}({\rho }_{AB})$ exceeds ${{\mathcal{B}}}_{k}$ for 1 ≤ *k* ≤ *d* − 1, then the Schmidt number of *ρ*_*A* *B*_ must be at least *k* + 1. For a given set of local measurement bases ${\{{\{| {e}_{a}^{z}\left.\right\rangle \}}_{a = 0}^{d-1}\}}_{z = 1}^{m}$, parties A and B can maximize the certified Schmidt number by choosing *U* (their relative reference frame) that maximizes ${{\mathcal{S}}}_{d}^{(m)}({\rho }_{AB})$ since the upper bound in eq. (4) is independent of *U*. Notice that if all the measurement bases are MUBs, i.e., $| \left\langle \right.{e}_{a}^{z}| {e}_{{a}^{{\prime} }}^{{z}^{{\prime} }}\left.\right\rangle {| }^{2}=\frac{1}{d}\,\forall \,a,{a}^{{\prime} },z\ne {z}^{{\prime} }$, then ${\mathcal{T}}({\mathcal{C}})=1$ and the bound in eq. (4) coincides with the one in ref. ^23^. Since ${\mathcal{T}}({\mathcal{C}})\ge 1$ for any *m* ≥ 2, we immediately arrive at Corollary 1.

#### Corollary 1

The bound ${{\mathcal{B}}}_{k}$ in eq. (4) is the tightest for all *k* < *d* when the *m* measurement bases are MUBs.

In case we only have access to the set of maximum and minimum overlaps $\overline{{\mathcal{C}}}$ instead of all the overlaps ${\mathcal{C}}$, we can still bound the Schmidt number and the entanglement fidelity by loosening the bounds in Theorem 1. By maximizing the term ${\sum }_{a,{a}^{{\prime} }}| \left\langle \right.{e}_{a}^{z}| {e}_{{a}^{{\prime} }}^{{z}^{{\prime} }}\left.\right\rangle {| }^{4}$ in eq. (4) such that the overlaps are compatible with $\overline{{\mathcal{C}}}$, we obtain the following lemma which is proven in the Methods.

#### Lemma 1

For any bipartite state *ρ*_*A* *B*_ of equal local dimension *d* and Schmidt number at most *k*, it holds that

6 $${{\mathcal{S}}}_{d}^{(m)}({\rho }_{AB})\le \frac{k(m-\overline{{\mathcal{T}}}(\overline{{\mathcal{C}}}))}{d}+\overline{{\mathcal{T}}}(\overline{{\mathcal{C}}})=:{\overline{{\mathcal{B}}}}_{k},$$

where the upper bound ${\overline{{\mathcal{B}}}}_{k}$ depends on *d*, *k*, the number of measurement bases *m*, and a quantity $\overline{{\mathcal{T}}}(\overline{{\mathcal{C}}})$ which depends on the set of minimum and maximum bases overlaps. More specifically, $\overline{{\mathcal{T}}}(\overline{{\mathcal{C}}}):= \min \{\overline{\lambda }(\overline{{\mathcal{C}}}),m\}$, $\overline{\lambda }(\overline{{\mathcal{C}}}):= \frac{1}{2}\left(1+\sqrt{1+2d{\sum }_{z\ne {z}^{{\prime} }}\overline{G}({c}_{\max }^{z,{z}^{{\prime} }},{c}_{\min }^{z,{z}^{{\prime} }})}\right)\ge 1$, $\overline{G}({c}_{\max }^{z,{z}^{{\prime} }},{c}_{\min }^{z,{z}^{{\prime} }}):= 1-(d+1){c}_{\min }^{z,{z}^{{\prime} }}+{\Omega }^{z,{z}^{{\prime} }}$, ${\Omega }^{z,{z}^{{\prime} }}:={L}^{z,{z}^{{\prime} }}{({c}_{\max }^{z,{z}^{{\prime} }})}^{2}+(d-{L}^{z,{z}^{{\prime} }}-1){({c}_{\min }^{z,{z}^{{\prime} }})}^{2}\;+\;{[1-{L}^{z,{z}^{{\prime} }}{c}_{\max }^{z,{z}^{{\prime} }}-(d-{L}^{z,{z}^{{\prime} }}-1){c}_{\min }^{z,{z}^{{\prime} }}]}^{2}$, and

7 $${L}^{z,{z}^{{\prime} }}:= \left\{\begin{array}{ll}\left\lfloor \frac{1-{c}_{\min }^{z,{z}^{{\prime} }}d}{{c}_{\max }^{z,{z}^{{\prime} }}-{c}_{\min }^{z,{z}^{{\prime} }}}\right\rfloor \quad &\,{\text{if}}\,\,\,{c}_{\max }^{z,{z}^{{\prime} }} > {c}_{\min }^{z,{z}^{{\prime} }},\\ d\quad &\,{\text{if}}\,\,\,{c}_{\max }^{z,{z}^{{\prime} }}={c}_{\min }^{z,{z}^{{\prime} }}.\end{array}\right.$$

Furthermore, the entanglement fidelity of *ρ*_*A* *B*_ can be lower bounded as follows:

8 $${\mathcal{F}}({\rho}_{AB}) \ge {\max} \left \{0,\frac{\mathop{\mathcal{S}}\nolimits_d^{(m)}({\rho }_{AB})-{\overline{\mathcal{T}}}({\overline{\mathcal{C}}})}{m-{\overline{\mathcal{T}}}({\overline{\mathcal{C}}})}\right\}=:{{\overline{\mathcal{F}}}}_{m}.$$

Similar to Theorem 1, if ${{\mathcal{S}}}_{d}^{(m)}({\rho }_{AB}) > {\overline{{\mathcal{B}}}}_{k}$, the Schmidt number of *ρ*_*A* *B*_ must be at least *k* + 1. Furthermore, if all the measurement bases are MUBs, i.e., ${c}_{\max }^{z,{z}^{{\prime} }}={c}_{\min }^{z,{z}^{{\prime} }}=\frac{1}{d}\,\forall \,z,{z}^{{\prime} }$, then $\overline{{\mathcal{T}}}(\overline{{\mathcal{C}}})=1$ and ${\overline{{\mathcal{B}}}}_{k}$ coincides with the bound in ref. ^23^. Since $\overline{{\mathcal{T}}}(\overline{{\mathcal{C}}})\ge 1$, MUBs give the tightest bounds ${\overline{{\mathcal{B}}}}_{k}$.

### Examples of witness violation

To illustrate that our method can verify Schmidt numbers and lower bound the entanglement fidelity, we first apply our witness and the fidelity bound to a standard benchmark for entanglement witnesses, i.e., isotropic states ${\rho }_{A\,B}^{{\rm{iso}}}=(1-p)| {\Phi }_{d}^{+}\left.\right\rangle \left\langle \right.{\Phi }_{d}^{+}| +\frac{p}{{d}^{2}}{{\mathbb{1}}}_{{d}^{2}}$, whose Schmidt number is *k* + 1 if and only if the white-noise ratio *p* satisfies $\frac{d(d-k-1)}{{d}^{2}-1}\le p < \frac{d(d-k)}{{d}^{2}-1}=:{p}_{\,\text{iso}\,}^{(k)}$^24^. We compare this with the noise that our witness can tolerate until we can no longer witness the actual Schmidt number of ${\rho }_{AB}^{{\rm{iso}}}$. Since

9 $${{\mathcal{S}}}_{d}^{(m)}({\rho }_{AB}^{{\rm{iso}}})=p\frac{m}{d}+(1-p)m,$$

for ${{\mathcal{S}}}_{d}^{(m)}({\rho }_{AB}^{{\rm{iso}}})$ to exceed the bound ${{\mathcal{B}}}_{k}$ in Theorem 1, the white-noise ratio must satisfy

10 $$p < \frac{(m-{\mathcal{T}}({\mathcal{C}}))(d-k)}{m(d-1)}=:{p}_{c,m}^{(k)}.$$

In the case when *d* + 1 MUBs exist and *m* = *d* + 1, we see that ${p}_{c,m}^{(k)}={p}_{\,\text{iso}\,}^{(k)}$ for all *k*.

**Fig. 1:**
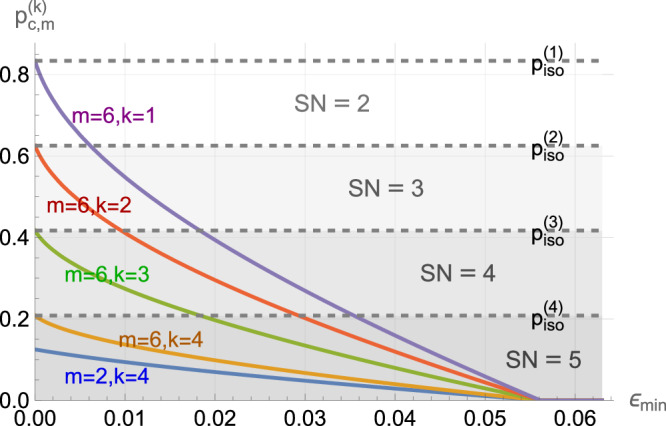
White-noise tolerance of our Schmidt-number witness. The upper bounds of the white-noise ratio, ${p}_{c,m}^{(k)}$ in eq. (10), for witnessing Schmidt number *k* + 1 versus ${\epsilon }_{\min }:= 1/d-{c}_{\min }$ in local dimension *d* = 5, where we set ${c}_{\max }=1-(d-1){c}_{\min }$. When ${\epsilon }_{\min }=0$ (i.e., ${c}_{\min }=1/d$ for MUBs), ${p}_{c,m}^{(k)}$ reaches its maximum, (*m* − 1)(*d* − *k*)/[*m*(*d* − 1)]. When *m* = *d* + 1 = 6, it coincides with ${p}_{\,\text{iso}\,}^{(k)}$, the maximum white-noise ratio for ${\rho }_{A\,B}^{{\rm{iso}}}$ having Schmidt number *k* + 1. The noise tolerance of the witness is higher for larger *m* or smaller *k* but reduces as ${\epsilon }_{\min }$ increases, and eventually, when ${\epsilon }_{\min }\ge 1/5-(7-\sqrt{17})/20\approx 0.0562$, we cannot witness non-trivial Schmidt numbers of ${\rho }_{AB}^{{\rm{iso}}}$.

**Fig. 2:**
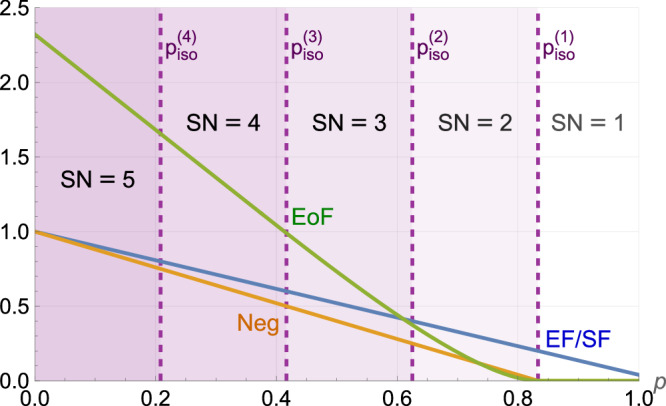
Comparing other standard measures of bipartite entanglement with the Schmidt number. The entanglement of formation (EoF), the entanglement fidelity/singlet fraction (EF/SF), the negativity (Neg), and the exact Schmidt number (SN) of the isotropic state ${\rho }_{AB}^{{\rm{iso}}}$ of *d* = 5 are plotted for different white-noise ratios *p*.

Suppose that we have the worst possible choice of measurement bases for a given ${c}_{\min }:= \mathop{\min }\nolimits_{z,{z}^{{\prime} }}{c}_{\min }^{z,{z}^{{\prime} }}$ such that ${c}_{\min }={c}_{\min }^{z,{z}^{{\prime} }}$ and ${c}_{\max }^{z,{z}^{{\prime} }}=1-(d-1){c}_{\min }=:{c}_{\max }$ for all $z,{z}^{{\prime} }$ (As it is harder to witness a state to have Schmidt number *k* + 1 with a larger bound ${{\mathcal{B}}}_{k}$, this is the worst-case bases choice for a given ${c}_{\min }=\mathop{\min }\nolimits_{z,{z}^{{\prime} }}{c}_{\min }^{z,{z}^{{\prime} }}$ because (i) it gives the maximal value allowed for ${c}_{\max }=\mathop{\max }\nolimits_{z,{z}^{{\prime} }}{c}_{\max }^{z,{z}^{{\prime} }}$ such that ${\sum }_{{a}^{{\prime} }}| \langle {e}_{a}^{z}| {e}_{{a}^{{\prime} }}^{{z}^{{\prime} }}\rangle {| }^{2}=1$ holds for all *a*, and (ii) ${\mathcal{T}}({\mathcal{C}})$ (and therefore ${{\mathcal{B}}}_{k}$) increases with ${c}_{\max }$ for a fixed ${c}_{\min }$ due to the enlargement of the feasible set of the optimization problem in Proposition 3). We can use the upper bound in Lemma 1 by replacing ${\mathcal{T}}({\mathcal{C}})$ in eq. (10) with $\overline{{\mathcal{T}}}(\overline{{\mathcal{C}}})$, where $\overline{\lambda }(\overline{{\mathcal{C}}})=\frac{1}{2}(1+\sqrt{1+2dm(m-1)\overline{G}({c}_{\max },{c}_{\min })})$ in eq. (6). To verify that our state has Schmidt number at least *k* + 1, the bound ${\overline{{\mathcal{B}}}}_{k}$ must be violated. In Fig. 1, the white-noise thresholds ${p}_{c,m}^{(k)}$ for witnessing the Schmidt number of ${\rho }_{AB}^{{\rm{iso}}}$ in *d* = 5 to be at least *k* + 1 with *m* measurement bases are plotted against the parameter ${\epsilon }_{\min }:= \frac{1}{d}-{c}_{\min }$, which quantifies the deviation of the measurement bases from MUBs. Note that the maximum value that ${c}_{\min }$ can take is $\frac{1}{d}$ in order for ${\sum }_{{a}^{{\prime} }}| \left\langle \right.{e}_{a}^{z}| {e}_{{a}^{{\prime} }}^{{z}^{{\prime} }}\left.\right\rangle {| }^{2}=1$ to hold for all *a*, *z* and ${z}^{{\prime} }$, so ${\epsilon }_{\min }\in [0,\frac{1}{d}]$. When ${\epsilon }_{\min }=0$, corresponding to measurements in MUBs, ${p}_{c,m}^{(k)}$ attains its maximum. In general, the witness can be violated under more white noise for smaller *k*. In addition, as ${p}_{c,m}^{(k)}$ goes to zero when $\overline{\lambda }(\overline{{\mathcal{C}}})=m$, we see that whenever

11 $${c}_{\min }\le \frac{3d-1-\sqrt{{d}^{2}+10d-7}}{2d(d-1)},$$

we cannot witness any Schmidt number 2 ≤ *k* + 1 ≤ *d* of the state ${\rho }_{AB}^{{\rm{iso}}}$ as ${p}_{c,m}^{(k)}=0$ for all *m* ≥ 2. To see how the Schmidt number is related to other standard measures of bipartite entanglement, we also compare the entanglement of formation (EoF) (see eq. (4.2) of ref. ^49^), the entanglement fidelity/singlet fraction (EF/SF), and the negativity (see eq. (26) of ref. ^50^) with the exact Schmidt number (SN) of the isotropic state of *d* = 5 for different white-noise ratios *p* in Fig. 2.

In the Supplementary Information, we provide more elaborate analyses of our witness when applied to isotropic states. First, we show that the entanglement fidelity of ${\rho }_{AB}^{{\rm{iso}}}$ satisfies eq. (5) in Theorem 1. Then, we provide an example suggesting that our Schmidt-number witness can tolerate less bases bias in larger local dimensions. Finally, we compare the white-noise tolerance of our witness and our lower bound on the entanglement fidelity with the counterparts from ref. ^10^.

To give a more comprehensive picture, we provide another example, i.e., the purified thermal states with white noise, in the Supplementary Information, to demonstrate that our method also works in cases where the eigenvalues of the single-party reduced states are not degenerate and to compare the white-noise tolerance of our witness with that of ref. ^10^ in such cases. We also show that adding a third basis that is slightly biased with respect to two mutually unbiased measurement bases can increase our witness’ tolerance to white noise in this example. On the other hand, adding a basis that is too biased with respect to the other bases could worsen our witnesses’ performance due to an increased upper bound ${{\mathcal{B}}}_{k}$ (${\overline{{\mathcal{B}}}}_{k}$) in Theorem 1 (Lemma 1). We summarize this observation in the following remark.

#### Remark 1

There exist scenarios where an additional measurement basis improves the noise tolerance of our Schmidt-number witness. However, the opposite can also occur for certain choices of bases. Therefore, in order to witness the highest Schmidt number of a state, one should apply the witness inequality in Theorem 1 or Lemma 1 to all subsets of the total set of ${m}^{{\prime} }$ available measurement bases and find the largest *k* such that ${{\mathcal{S}}}_{d}^{(m)}(\rho ) > {{\mathcal{B}}}_{k}$ or ${\overline{{\mathcal{B}}}}_{k}$ when evaluated over all subsets of *m* chosen bases for all $m\in \{2,\ldots ,{m}^{{\prime} }\}$.

The intuition behind Remark 1 is that one can potentially certify a higher Schmidt number by post-selecting a subset of the total measurement data that achieves the optimal balance between showing the strongest measurement correlations and minimizing bias in the measurement bases. In practice, this can be realized easily by using as many local orthogonal measurement bases as possible for both parties and then calculating both the sum of expectation values ${{\mathcal{S}}}_{d}^{(m)}(\rho )$ and the bounds ${{\mathcal{B}}}_{k}$ or ${\overline{{\mathcal{B}}}}_{k}$ for each subset of measurement bases, using the corresponding subset of the full measurement data.

As a further remark, we observe that in *d* = 6, adding a fourth basis to a set with three MUBs decreases the noise tolerance for witnessing Schmidt numbers in isotropic states for a wide range of choices for the additional basis. Since it is widely believed that the maximum number of MUBs in *d* = 6 is 3^38^, this observation could indicate that our witness performs best when the local measurement bases consist only of the maximal set of MUBs in the given local dimension and no other bases.

### Implication of concentration of measure

We have seen from Corollary 1 that measuring in MUBs will give the best Schmidt-number witness. However, requiring all local measurement bases to be MUBs is experimentally demanding as it requires precise control over the relative phases among all measurement bases. In light of this practical difficulty, it is natural to ask, how likely will a set of measurement bases chosen uniformly at random in ${{\mathbb{C}}}^{d}$ be close to being mutually unbiased? Using Lévy’s lemma^34–36^, a result from concentration of measure, we show that the likelihood of any two randomly chosen orthonormal bases to be biased decreases exponentially with the dimension *d*, i.e., for *ϵ* > 0,

12 $$\,\text{Pr}\,\left\{\left| {\left| \left\langle \right.{e}_{a}^{z}| {e}_{{a}^{{\prime} }}^{{z}^{{\prime} }}\left.\right\rangle \right| }^{2}-\frac{1}{d}\right| > \epsilon \right\}\le 2\exp \left(-\frac{d{\epsilon }^{2}}{18{\pi }^{3}\ln 2}\right),$$

for all $a,{a}^{{\prime} }$ and $z\ne {z}^{{\prime} }$. Therefore, in large dimensions *d*, random measurement bases are likely to be sufficient for our method to witness high-dimensional entanglement. The proof of eq. (12) can be found in Sec. S.III of the Supplementary Information.

### Maximal number of orthonormal bases

Intuitively, one cannot construct arbitrarily many orthonormal bases when the maximal and minimal bases overlaps are specified. For example, it was known that there cannot be more than *d* + 1 MUBs in ${{\mathbb{C}}}^{d}$ (where ${c}_{\max }^{z,{z}^{{\prime} }}={c}_{\min }^{z,{z}^{{\prime} }}=\frac{1}{d}$)^48^. By making a connection to the Welch bounds^37^, we can upper bound the number of orthonormal bases using the function $\lambda ({\mathcal{C}})$ defined in Theorem 1 such that

13 $$m\le \frac{d+1}{2}\left(1+\sqrt{1+\frac{8\lambda ({\mathcal{C}})(\lambda ({\mathcal{C}})-1)}{{d}^{2}-1}}\right)=:{\overline{m}}_{d},$$

which is proven in Sec. S.IV of the Supplementary Information. For MUBs, we have $\lambda ({\mathcal{C}})=1$, so eq. (13) becomes *m* ≤ *d* + 1, which agrees with the known upper bound^48^. In general, the bound does not imply the existence of ${\overline{m}}_{d}$ orthonormal bases. For instance, the existence of *d* + 1 MUBs for non-prime-power dimensions *d* is still an open problem^51^. In Sec. S.I.1 of the Supplementary Information, we use this bound to show that eq. (5) is satisfied for our example.

### Simple construction of three MUBs in any dimension

As a by-product of investigating the use of AMUBs for witnessing high-dimensional entanglement (see Sec. S.VI of the Supplementary Information), we discover a construction of three MUBs that has a simple analytic form and works for any dimension $d\in {\mathbb{N}}$. The nice feature about this is that it does not rely on (the tensor products of) the Wootters–Fields bases^48^, which inevitably requires knowing the prime factorization of the dimension. Since factorizing a large integer is assumed to be hard (at least before any quantum device can properly implement Shor’s algorithm)^52^ and the description of the Wootters–Fields bases can be non-trivial for large prime powers^48^, constructing MUBs with the tensor products of the Wootters–Fields bases will require a certain amount of computational overhead, whereas our construction does not suffer from these problems despite having only three MUBs.

The explicit form of our three-MUBs construction is stated in the following lemma and its proof can be found in Sec. S.V of the Supplementary Information.

#### Lemma 2

For any $d\in {\mathbb{N}}$, the three orthonormal bases ${\{| {e}_{a}^{1}\left.\right\rangle = | a\left.\right\rangle \}}_{a = 0}^{d-1}$, ${\{| {e}_{a}^{2}\left.\right\rangle \}}_{a = 0}^{d-1}$, and ${\{| {e}_{a}^{3}\left.\right\rangle \}}_{a = 0}^{d-1}$, with

14a $$| {e}_{a}^{2}\left.\right\rangle =\frac{1}{\sqrt{d}}\mathop{\sum }\limits_{j=0}^{d-1}{e}^{i2\pi \left[\frac{aj}{d}+f(j)\right]}| j\left.\right\rangle ,$$

14b $$| {e}_{a}^{3}\left.\right\rangle =\frac{1}{\sqrt{d}}\mathop{\sum }\limits_{j=0}^{d-1}{e}^{i2\pi \left[\frac{(d-{p}^{r}){j}^{2}}{2d}+\frac{aj}{d}+f(j)\right]}| j\left.\right\rangle ,$$

where *f* is any real-valued function, $r\in {\mathbb{N}}\cup \{0\}$, and *p* is any odd prime such that *gcd*(*d*, *p*) = 1 and *d* > *p*^*r*^, are mutually unbiased. The simplest example would be having *p*^*r*^ = 1.

Since the function *f* (and to some degree, *p*^*r*^) can be chosen freely, it can be optimized such that the relative phases in eqs. (14a) and (14b) are more easily realizable for different experimental setups. This flexibility is particularly useful in cases where the dimension of the Hilbert space in which the experiment operates can change over time (e.g., in scenarios where one has a dynamical encoding protocol that encodes information in different subspaces of the physical system at different times to protect against some time-dependent noise which affects various parts of the system in distinct ways) or the experiment has to measure different subsystems with distinct subspace dimensions at different times, as recalibration of the relative phases of our bases may require less drastic changes to the setup than using (the tensor product of) the Wootters-Fields bases. For the simplest example, if one needs to encode information or measure in 3 MUBs and the corresponding Hilbert space switches from dimension 7 to 6, then our simple construction only requires a slight change in the phases (especially with the free choices of *f*(*j*) and *p*^*r*^ for compensation), whereas using the Wootters-Fields bases will need to switch from the prime-dimension Wootters-Fields basis for *d* = 7 to the tensor products of the Wootters-Fields bases of dimensions 2 and 3, which introduce more drastic changes to the relative phases. For large *d*, the change to the relative phases can be even more drastic.

## Discussion

Our results provide a fresh perspective on the longstanding problem of detecting entanglement using only a few, potentially restricted, measurement settings. Specifically, we introduced a family of Schmidt-number witnesses based on correlations in at least two coordinated local orthonormal bases that can be chosen arbitrarily. We established analytic upper bounds for the corresponding witness operators when evaluated on any bipartite state with a Schmidt number of at most *k*. The main advantage of our method is that the bounds depend solely on the absolute values of the overlaps between different measurement bases, but not on their relative phases, which are often inaccessible in experiments. These features of our witness simplify experimental requirements for certifying high-dimensional entanglement across many platforms. We demonstrated the effectiveness of our witness with two-qudit isotropic states and noisy purified thermal states. We also discussed the use of random measurement bases to witness Schmidt numbers. Finally, we compare our method with various existing approaches for certifying high-dimensional entanglement in Table 1.

As Corollary 1 suggests, one should aim at locally measuring in as many MUBs as possible to get the best performance of our witness. Sadly, the total number of MUBs is unknown for dimensions that are not prime powers^51^. In fact, given the prime factorization of the dimension $d={\prod }_{j}{p}_{j}^{{n}_{j}}$ with ${p}_{j}^{{n}_{j}} < {p}_{j+1}^{{n}_{j+1}}\,\forall \,j$, (tensor products of) the Wootters–Fields construction only guarantees ${p}_{1}^{{n}_{1}}+1$ MUBs to exist^38^. Alternatively, if one can measure in bases that are nearly mutually unbiased, then one can construct *d* + 1 AMUBs for any dimension *d*^40^. We constructed Schmidt-number witnesses based on the AMUBs proposed in ref. ^40^, but we did not observe any advantage of measuring in 4 ≤ *m* ≤ *d*  + 1 AMUBs compared to measuring in three MUBs in the non-prime-powered dimensions *d* = 6, 10, 14, 22 (see Sec. S.VI of the Supplementary Information). The discovery of the simple three-MUBs construction in any dimension (Lemma 2) by modifying the AMUBs construction in ref. ^40^ suggests that there could be other constructions of AMUBs that are more suitable for witnessing Schmidt numbers and we leave finding such bases as an open problem. Furthermore, given the flexibility of our three-MUBs construction, it may even contribute to answering a longstanding mathematical problem: Are there more than three MUBs in even, non-prime-power dimensions such as *d* = 6?

## Methods

### Proof of Theorem 1

To prove Theorem 1, we start by stating the following propositions that we will need in the main proof.

#### Proposition 1

It holds that $A\otimes {{\mathbb{1}}}_{d}| {\Phi }_{d}^{+}\left.\right\rangle ={{\mathbb{1}}}_{d}\otimes {A}^{T}| {\Phi }_{d}^{+}\left.\right\rangle$ for all $A\in M({{\mathbb{C}}}^{d})$.

#### Proof

Let $$A=\mathop{\sum }\nolimits_{i,j = 0}^{d-1}{A}_{ij}| i\left.\right\rangle \left\langle \right.j| $$. Then,

15a $$\begin{array}{rcl}A\otimes {{\mathbb{1}}}_{d}| {\Phi }_{d}^{+}\left.\right\rangle &=&\frac{1}{\sqrt{d}}\mathop{\sum }\limits_{i,j,k=0}^{d-1}{A}_{ij}| i\left.\right\rangle \left\langle \right.j| k\left.\right\rangle \otimes | k\left.\right\rangle \\ &=&\frac{1}{\sqrt{d}}\mathop{\sum }\limits_{i,j=0}^{d-1}{A}_{ij}| i\left.\right\rangle \otimes | j\left.\right\rangle ,\end{array}$$

15b $$\begin{array}{rcl}{{\mathbb{1}}}_{d}\otimes {A}^{T}| {\Phi }_{d}^{+}\left.\right\rangle &=&\frac{1}{\sqrt{d}}\mathop{\sum }\limits_{i,j,k=0}^{d-1}{A}_{ji}| k\left.\right\rangle \otimes | i\left.\right\rangle \left\langle \right.j| k\left.\right\rangle \\ &=&\frac{1}{\sqrt{d}}\mathop{\sum }\limits_{i,j=0}^{d-1}{A}_{ji}| j\left.\right\rangle \otimes | i\left.\right\rangle ,\end{array}$$

so we indeed have $A\otimes {{\mathbb{1}}}_{d}| {\Phi }_{d}^{+}\left.\right\rangle ={{\mathbb{1}}}_{d}\otimes {A}^{T}| {\Phi }_{d}^{+}\left.\right\rangle$. □

#### Proposition 2

Let $| \psi \left.\right\rangle$ and $| \phi \left.\right\rangle$ be normalized states. Then, the eigenvalues of $| \psi \left.\right\rangle \left\langle \right.\phi | +| \phi \left.\right\rangle \left\langle \right.\psi |$ are upper bounded by $| \left\langle \right.\psi | \phi \left.\right\rangle | +1$ and lower bounded by $-(| \left\langle \right.\psi | \phi \left.\right\rangle | +1)$.

#### Proof

Let $| \phi \left.\right\rangle =a| \psi \left.\right\rangle +b| {\psi }^{\perp }\left.\right\rangle$ such that ∣*a*∣^2^ + ∣*b*∣^2^ = 1, $\left\langle \right.\psi | {\psi }^{\perp }\left.\right\rangle =0$, and $\left\langle \right.\psi | \psi \left.\right\rangle =1=\left\langle \right.{\psi }^{\perp }| {\psi }^{\perp }\left.\right\rangle$. Then, $| \psi \left.\right\rangle \left\langle \right.\phi | +| \phi \left.\right\rangle \left\langle \right.\psi | =(a+{a}^{* })| \psi \left.\right\rangle \left\langle \right.\psi | +{b}^{* }| \psi \left.\right\rangle \left\langle \right.{\psi }^{\perp }| +b| {\psi }^{\perp }\left.\right\rangle \left\langle \right.\psi |$, which has eigenvalues ${\lambda }_{\pm }=\,\text{Re}(a)\pm \sqrt{1-\text{Im}\,{(a)}^{2}}$. Since Re(*a*) ≤ ∣*a*∣ and $\sqrt{1-\,\text{Im}\,{(a)}^{2}}\le 1$, we have $| {\lambda }_{\pm }| \le | a| +1=| \left\langle \right.\psi | \phi \left.\right\rangle | +1$. □

We are now ready to state the formal proof of Theorem 1 which follows similar arguments for proving Result 1 in ref. ^23^ except here, the reference frame of party B relative to party A’s is fixed by an arbitrary unitary and we cannot assume that $| \left\langle \right.{e}_{a}^{z}| {e}_{{a}^{{\prime} }}^{{z}^{{\prime} }}\left.\right\rangle {| }^{2}=\frac{1}{d}$ for all $z\ne {z}^{{\prime} }$.

#### Proof of Theorem 1

Let us define the witness operator

16 $$W=\mathop{\sum }\limits_{z=1}^{m}\mathop{\sum }\limits_{a=0}^{d-1}| {e}_{a}^{z}\left.\right\rangle \left\langle \right.{e}_{a}^{z}| \otimes | {\tilde{e}}_{a}^{z*} \left.\right\rangle \left\langle \right.{\tilde{e}}_{a}^{z*} |$$

so that $\,\text{Tr}\,(W{\rho }_{AB})={{\mathcal{S}}}_{d}^{(m)}({\rho }_{AB})$, where $|{\tilde{e}}_{a}^{z*} \left.\right\rangle =U| {e}_{a}^{z*} \left.\right\rangle$ and *U* is a fixed unitary that is the same for all *a* and *z*. Via the definition of $| {\tilde{e}}_{a}^{z*} \left.\right\rangle$, we have $\mathop{\sum }\nolimits_{a = 0}^{d-1}(| {e}_{a}^{z}\left.\right\rangle \left\langle \right.{e}_{a}^{z}| \otimes | {\tilde{e}}_{a}^{z*} \left.\right\rangle \left\langle \right.{\tilde{e}}_{a}^{z*} | )({{\mathbb{1}}}_{d}\otimes U)| {\Phi }_{d}^{+}\left.\right\rangle =\mathop{\sum }\nolimits_{a = 0}^{d-1}(| {e}_{a}^{z}\left.\right\rangle \left\langle \right.{e}_{a}^{z}| \otimes U| {e}_{a}^{z*} \left.\right\rangle \left\langle \right.{e}_{a}^{z*} | )| {\Phi }_{d}^{+}\left.\right\rangle =\mathop{\sum }\nolimits_{a = 0}^{d-1}| {e}_{a}^{z}\left.\right\rangle \left\langle \right.{e}_{a}^{z}| {e}_{a}^{z}\left.\right\rangle \left\langle \right.{e}_{a}^{z}| \otimes U| {\Phi }_{d}^{+}\left.\right\rangle =({{\mathbb{1}}}_{d}\otimes U)| {\Phi }_{d}^{+}\left.\right\rangle =:| {\widetilde{\Phi }}_{d}^{+}\left.\right\rangle$, where we have used Proposition 1 in the second step. Hence, we have $W| {\widetilde{\Phi }}_{d}^{+}\left.\right\rangle =m| {\widetilde{\Phi }}_{d}^{+}\left.\right\rangle$. Since *W* is positive semi-definite, it has a spectral decomposition: $W=m| {\widetilde{\Phi }}_{d}^{+}\left.\right\rangle \left\langle \right.{\widetilde{\Phi }}_{d}^{+}| +\mathop{\sum }\nolimits_{i = 2}^{{d}^{2}}{\lambda }_{i}| {\lambda }_{i}\left.\right\rangle \left\langle \right.{\lambda }_{i}|$ where all normalized eigenvectors $| {\lambda }_{i}\left.\right\rangle$ are orthogonal to $| {\widetilde{\Phi }}_{d}^{+}\left.\right\rangle$.

Next, we will derive an upper bound for all the eigenvalues *λ*_*i*_ with *i* = 2, …, *d*^2^. To do this, we consider the operator

17 $$\begin{array}{ll}{W}^{2}=W+\sum\limits_{z\ne {z}^{{\prime} }}\sum\limits_{a,{a}^{{\prime} }}| \left\langle \right.{e}_{a}^{z}| {e}_{{a}^{{\prime} }}^{{z}^{{\prime} }}\left.\right\rangle {| }^{2}| {e}_{a}^{z}{\tilde{e}}_{a}^{z*} \left.\right\rangle \left\langle \right.{e}_{{a}^{{\prime} }}^{{z}^{{\prime} }}{\tilde{e}}_{{a}^{{\prime} }}^{{z}^{{\prime} }*} | \\ \qquad=W+\sum\limits_{z\ne {z}^{{\prime} }}{c}_{\min }^{z,{z}^{{\prime} }}{{\mathcal{T}}}_{1}^{z,{z}^{{\prime} }}+{{\mathcal{T}}}_{2},\end{array}$$

with ${{\mathcal{T}}}_{1}^{z,{z}^{{\prime} }}={\sum }_{a,{a}^{{\prime} }}| {e}_{a}^{z}{\tilde{e}}_{a}^{z*} \left.\right\rangle \left\langle \right.{e}_{{a}^{{\prime} }}^{{z}^{{\prime} }}{\tilde{e}}_{{a}^{{\prime} }}^{{z}^{{\prime}* }} | =d| {\widetilde{\Phi }}_{d}^{+}\left.\right\rangle \left\langle \right.{\widetilde{\Phi }}_{d}^{+}|$ since *V* ⊗ V^*^ with $V=\mathop{\sum }\nolimits_{j = 0}^{d-1}| {e}_{j}^{z}\left.\right\rangle \left\langle \right.j|$ is a symmetry of $| {\Phi }_{d}^{+}\left.\right\rangle$ by Proposition 1, and

18 $$\begin{array}{ll}{{\mathcal{T}}}_{2}=\sum\limits_{z\ne {z}^{{\prime} }}\sum\limits_{a,{a}^{{\prime} }}\left(| \left\langle \right.{e}_{a}^{z}| {e}_{{a}^{{\prime} }}^{{z}^{{\prime} }}\left.\right\rangle {| }^{2}-{c}_{\min }^{z,{z}^{{\prime} }}\right)\left| {e}_{a}^{z}{\tilde{e}}_{a}^{z*} \left.\right\rangle \left\langle \right.{e}_{{a}^{{\prime} }}^{{z}^{{\prime} }}{\tilde{e}}_{{a}^{{\prime} }}^{{z}^{{\prime} }*} \right| \\ \quad\,=\sum\limits_{z\ne {z}^{{\prime} }}\sum\limits_{a,{a}^{{\prime} }}\left(| \left\langle \right.{e}_{a}^{z}| {e}_{{a}^{{\prime} }}^{{z}^{{\prime} }}\left.\right\rangle {| }^{2}-{c}_{\min }^{z,{z}^{{\prime} }}\right)\frac{1}{2}\left(| {e}_{a}^{z}{\tilde{e}}_{a}^{z*} \left.\right\rangle \left\langle \right.{e}_{{a}^{{\prime} }}^{{z}^{{\prime} }}{\tilde{e}}_{{a}^{{\prime} }}^{{z}^{{\prime} }*} | +\,\text{H.c.}\right),\end{array}$$

where we use the fact that $| \left\langle \right.{e}_{a}^{z}| {e}_{{a}^{{\prime} }}^{{z}^{{\prime} }}\left.\right\rangle | =| \left\langle \right.{e}_{{a}^{{\prime} }}^{{z}^{{\prime} }}| {e}_{a}^{z}\left.\right\rangle | \,\forall \,a,{a}^{{\prime} },z,{z}^{{\prime} }$ and “H.c.” stands for the Hermitian conjugate of the previous term. Then, we use (i) $| \left\langle \right.{e}_{a}^{z}| {e}_{{a}^{{\prime} }}^{{z}^{{\prime} }}\left.\right\rangle {| }^{2}-{c}_{\min }^{z,{z}^{{\prime} }}\ge 0$, (ii) $| {e}_{a}^{z}{\tilde{e}}_{a}^{z*} \left.\right\rangle \left\langle \right.{e}_{{a}^{{\prime} }}^{{z}^{{\prime}}}{\tilde{e}}_{{a}^{{\prime} }}^{{z}^{{\prime} }*} | +\,{\rm{H}}.{\rm{c}}.\,\le {\lambda}_{\max}(| {e}_{a}^{z}{\tilde{e}}_{a}^{z*} \left.\right\rangle \left\langle \right.{e}_{{a}^{{\prime}*}}^{{z}^{{\prime}}}{\tilde{e}}_{{a}^{{\prime} }}^{{z}^{{\prime} }*} | +\,{\rm{H}}.{\rm{c}}.\,){{\mathbb{1}}}_{{d}^{2}}$, where ${\lambda }_{\max }(A)$ denotes the largest eigenvalue of *A*, (iii) ${\lambda }_{\max }({\sum }_{i}{A}_{i})\le {\sum }_{i}{\lambda }_{\max }({A}_{i})$ for all ${A}_{i}\in \,\text{Herm}\,({{\mathbb{C}}}^{d})$^53^, and (iv) Proposition 2 to obtain

19 $$\begin{array}{ll}{{\mathcal{T}}}_{2}\le \frac{1}{2}\sum\limits_{z\ne {z}^{{\prime} }}\sum\limits_{a,{a}^{{\prime} }}\left(| \left\langle {e}_{a}^{z}| {e}_{{a}^{{\prime} }}^{{z}^{{\prime} }}\right\rangle {| }^{2}-{c}_{\min }^{z,{z}^{{\prime} }}\right) \,{\lambda }_{\max }\left(| {e}_{a}^{z}{\tilde{e}}_{a}^{z*} \left.\right\rangle \left\langle \right.{e}_{{a}^{{\prime} }}^{{z}^{{\prime} }}{\tilde{e}}_{{a}^{{\prime} }}^{{z}^{{\prime} }*} | +\,{\text{H}}.{\text{c}}.\,\right){{\mathbb{1}}}_{{d}^{2}}\\ \quad\le \frac{1}{2}\sum\limits_{z\ne {z}^{{\prime} }}\sum\limits_{a,{a}^{{\prime} }}\left(| \left\langle {e}_{a}^{z}| {e}_{{a}^{{\prime} }}^{{z}^{{\prime} }}\right\rangle {| }^{2}-{c}_{\min }^{z,{z}^{{\prime} }}\right)\left(| \left\langle {e}_{a}^{z}| {e}_{{a}^{{\prime} }}^{{z}^{{\prime} }} \right\rangle {| }^{2}+1\right){{\mathbb{1}}}_{{d}^{2}}.\end{array}$$

Finally, we use the equality $\mathop{\sum }\nolimits_{{a}^{{\prime} } = 0}^{d-1}| \left\langle \right.{e}_{a}^{z}| {e}_{{a}^{{\prime} }}^{{z}^{{\prime} }}\left.\right\rangle {| }^{2}=1$ to get

20 $$\begin{array}{l}{{\mathcal{T}}}_{2}\le \frac{1}{2}\sum\limits_{z\ne {z}^{{\prime} }}\left[\sum\limits_{a}(1-{c}_{\min }^{z,{z}^{{\prime} }}-{c}_{\min }^{z,{z}^{{\prime} }}d)+\sum\limits_{a,{a}^{{\prime} }}| \left\langle \right.{e}_{a}^{z}| {e}_{{a}^{{\prime} }}^{{z}^{{\prime} }}\left.\right\rangle {| }^{4}\right]{{\mathbb{1}}}_{{d}^{2}}\\\qquad=\frac{d}{2}\sum\limits_{z\ne {z}^{{\prime} }}\left[1-(d+1){c}_{\min }^{z,{z}^{{\prime} }}+\frac{1}{d}\sum\limits_{a,{a}^{{\prime} }}| \left\langle \right.{e}_{a}^{z}| {e}_{{a}^{{\prime} }}^{{z}^{{\prime} }}\left.\right\rangle {| }^{4}\right]{{\mathbb{1}}}_{{d}^{2}}\\ \qquad=:\frac{d}{2}\sum\limits_{z\ne {z}^{{\prime} }}{G}^{z,{z}^{{\prime} }}{{\mathbb{1}}}_{{d}^{2}}.\end{array}$$

After combining eqs. (17) and (20), we obtain

21 $${W}^{2}\le W+d\sum _{z\ne {z}^{{\prime} }}\left({c}_{\min }^{z,{z}^{{\prime} }}| {\widetilde{\Phi }}_{d}^{+}\left.\right\rangle \left\langle \right.{\widetilde{\Phi }}_{d}^{+}| +\frac{1}{2}{G}^{z,{z}^{{\prime} }}{{\mathbb{1}}}_{{d}^{2}}\right).$$

Since ${W}^{2}={m}^{2}| {\widetilde{\Phi }}_{d}^{+}\left.\right\rangle \left\langle \right.{\widetilde{\Phi }}_{d}^{+}| +\mathop{\sum }\nolimits_{i = 2}^{{d}^{2}}{\lambda }_{i}^{2}| {\lambda }_{i}\left.\right\rangle \left\langle \right.{\lambda }_{i}|$ and due to eq. (21), we have that ${\lambda }_{i}^{2}\le {\lambda }_{i}+\frac{d}{2}{\sum }_{z\ne {z}^{{\prime} }}{G}^{z,{z}^{{\prime} }}$ for *i* = 2, …, *d*^2^, which implies that ${\lambda }_{i}\le \frac{1}{2}\left(1+\sqrt{1+2d{\sum }_{z\ne {z}^{{\prime} }}{G}^{z,{z}^{{\prime} }}}\right)=:\lambda ({\mathcal{C}})$. Since ${G}^{z,{z}^{{\prime} }}\ge 0\,\forall \,z\ne {z}^{{\prime} }$ [see eq. (19)], $\lambda ({\mathcal{C}})\ge 1$. On the other hand, we know that ${\lambda }_{\max }(W)={\lambda }_{\max }(\mathop{\sum }\nolimits_{z = 1}^{m}\mathop{\sum }\nolimits_{a = 0}^{d-1}| {e}_{a}^{z}\left.\right\rangle \left\langle \right.{e}_{a}^{z}| \otimes | {\tilde{e}}_{a}^{z*} \left.\right\rangle \left\langle \right.{\tilde{e}}_{a}^{z*} | )\le {\sum }_{z}{\lambda }_{\max }({\sum }_{a}| {e}_{a}^{z}\left.\right\rangle \left\langle \right.{e}_{a}^{z}| \otimes | {\tilde{e}}_{a}^{z*} \left.\right\rangle \left\langle \right.{\tilde{e}}_{a}^{z*} | )=m$, so ${\lambda }_{i}\le {\mathcal{T}}({\mathcal{C}}):= \min \{\lambda ({\mathcal{C}}),m\}$. Therefore, with $W| {\widetilde{\Phi }}_{d}^{+}\left.\right\rangle =m| {\widetilde{\Phi }}_{d}^{+}\left.\right\rangle$, we have that

22 $$W\le (m-{\mathcal{T}}({\mathcal{C}}))| {\widetilde{\Phi }}_{d}^{+}\left.\right\rangle \left\langle \right.{\widetilde{\Phi }}_{d}^{+}| +{\mathcal{T}}({\mathcal{C}}){{\mathbb{1}}}_{{d}^{2}}.$$

Finally, we arrive at our bound in eq. (4) in Theorem 1:

23 $$\begin{array}{ll}{{\mathcal{S}}}_{d}^{(m)}({\rho }_{AB})\,=\,{\text{Tr}}\,(W{\rho }_{AB})& \le (m-{\mathcal{T}}({\mathcal{C}})){\mathcal{F}}({\rho }_{AB})+{\mathcal{T}}({\mathcal{C}})\\&\le \frac{k(m-{\mathcal{T}}({\mathcal{C}}))}{d}+{\mathcal{T}}({\mathcal{C}}),\end{array}$$

where we have used the fact that $\left\langle \right.{\widetilde{\Phi }}_{d}^{+}| {\rho }_{AB}| {\widetilde{\Phi }}_{d}^{+}\left.\right\rangle \le {\mathcal{F}}({\rho }_{AB})\le \frac{k}{d}$ for all bipartite state *ρ*_*A* *B*_ of equal local dimension *d* and Schmidt number at most *k*^24^. The fidelity lower bound in eq. (5) can also be obtained by rearranging the first line of eq. (23) and is non-trivial only if ${\mathcal{T}}({\mathcal{C}}) < {{\mathcal{S}}}_{d}^{(m)}({\rho }_{AB})$. Otherwise, we set ${{\mathcal{F}}}_{m}=0$ as ${\mathcal{F}}({\rho }_{AB})\ge 0$ always holds. □

### Proof of Lemma 1

To prove Lemma 1, we need the following proposition which is proven in “Proof of Proposition 3”.

#### Proposition 3

The optimal solution to the optimization problem: max $\mathop{\sum }\nolimits_{i = 1}^{{d}^{2}}{x}_{i}^{4}$ subject to $\mathop{\sum }\nolimits_{i = jd+1}^{jd+d}{x}_{i}^{2}=1$ for *j* = 0,…, *d* − 1, and $0\le \sqrt{{c}_{\min }}\le {x}_{i}\le \sqrt{{c}_{\max }}\le 1\,\forall \,i$ is $d\{L{c}_{\max }^{2}+(d-L-1){c}_{\min }^{2}+{[1-L{c}_{\max }-(d-L-1){c}_{\min }]}^{2}\}$, where

24 $$L=\left\{\begin{array}{ll}\left\lfloor \frac{1-{c}_{\min }d}{{c}_{\max }-{c}_{\min }}\right\rfloor \quad &\,{\text{if}}\,\,\,{c}_{\max }\, >\, {c}_{\min },\\ d\quad &\,{\text{if}}\,\,\,{c}_{\max }={c}_{\min }.\end{array}\right.$$

#### Proof of Lemma 1

We will prove that $\overline{{\mathcal{T}}}(\overline{{\mathcal{C}}})\ge {\mathcal{T}}({\mathcal{C}})$ for all bases overlaps ${\mathcal{C}}$ so that

25 $$\begin{array}{lll}{{\mathcal{S}}}_{d}^{(m)}({\rho }_{AB})\;\le \;{{\mathcal{B}}}_{k}=(1-\frac{k}{d}){\mathcal{T}}({\mathcal{C}})+\frac{km}{d}\\\qquad\qquad\quad\,\le \;(1-\frac{k}{d})\overline{{\mathcal{T}}}(\overline{{\mathcal{C}}})+\frac{km}{d}\end{array}$$

for all *k* ≤ *d* as in eq. (6). This boils down to showing that ${\sum }_{a,{a}^{{\prime} }}| \left\langle \right.{e}_{a}^{z}| {e}_{{a}^{{\prime} }}^{{z}^{{\prime} }}\left.\right\rangle {| }^{4}\le {\Omega }^{z,{z}^{{\prime} }}d$ which implies ${G}^{z,{z}^{{\prime} }}\le \overline{G}({c}_{\max }^{z,{z}^{{\prime} }},{c}_{\min }^{z,{z}^{{\prime} }})\,\forall \,z,{z}^{{\prime} }$.

For every pair of distinct bases $z,{z}^{{\prime} }$, we want to maximize ${\sum }_{a,{a}^{{\prime} }}| \left\langle \right.{e}_{a}^{z}| {e}_{{a}^{{\prime} }}^{{z}^{{\prime} }}\left.\right\rangle {| }^{4}$ given that ${\sum }_{{a}^{{\prime} }}| \left\langle \right.{e}_{a}^{z}| {e}_{{a}^{{\prime} }}^{{z}^{{\prime} }}\left.\right\rangle {| }^{2}=1\,\forall \,a$ and $0\le \sqrt{{c}_{\min }}\le | \left\langle \right.{e}_{a}^{z}| {e}_{{a}^{{\prime} }}^{{z}^{{\prime} }}\left.\right\rangle | \le \sqrt{{c}_{\max }}\le 1\,\forall \,a,{a}^{{\prime} }$. By setting ${x}_{i}=| \left\langle \right.{e}_{a}^{z}| {e}_{{a}^{{\prime} }}^{{z}^{{\prime} }}\left.\right\rangle |$ and $i=da+{a}^{{\prime} }+1$, we can apply Proposition 3 to obtain the maximum value, ${\Omega }^{z,{z}^{{\prime} }}d$. Hence, $\overline{{\mathcal{T}}}(\overline{{\mathcal{C}}})\ge {\mathcal{T}}({\mathcal{C}})$ and eq. (6) holds. Finally, the fidelity lower bound in eq. (8) can be obtained in a similar fashion as in the proof of Theorem 1. The bound is non-trivial only if $\overline{{\mathcal{T}}}(\overline{{\mathcal{C}}}) < {{\mathcal{S}}}_{d}^{(m)}({\rho }_{AB})$. Otherwise, we set ${\overline{{\mathcal{F}}}}_{m}=0$ since ${\mathcal{F}}({\rho }_{AB})\ge 0$ always holds. □

#### Proof of Proposition 3

A general constrained optimization problem can be written in the following form^54^.

##### **Problem 1**

Let $\vec {x}\in {{\mathbb{R}}}^{d}$. A constrained optimization problem can be written as

26 $$\begin{array}{ll}\,{\text{minimize}} \quad f(\vec {x})\\ \,{\rm{subject}}\, {\rm{to}} \quad {h}_{1}(\vec {x})=0,\ldots ,{h}_{m}(\vec {x})=0,\\\qquad\qquad\quad{g}_{1}(\vec {x})\le 0,\ldots ,{g}_{r}(\vec {x})\le 0,\end{array}$$

where *f*, *h*_*i*_, *g*_*j*_ are functions mapping from ${{\mathbb{R}}}^{d}$ to ${\mathbb{R}}$. The feasible set $X\subset {{\mathbb{R}}}^{d}$ is composed of all the $\vec {x}\in {{\mathbb{R}}}^{d}$ that satisfy all the equality and inequality constraints. The associated Lagrangian of the problem is given by

27 $$L(\vec {x},\vec{\lambda },\vec{\mu })=f(\vec {x})+\mathop{\sum }\limits_{i=1}^{m}{\lambda }_{i}{h}_{i}(\vec {x})+\mathop{\sum }\limits_{j=1}^{r}{\mu }_{j}{g}_{j}(\vec {x}),$$

where ${\lambda }_{i},{\mu }_{j}\in {\mathbb{R}}$ are Lagrange multipliers.

It is useful to give the following definitions which we quote directly from ref. ^54^ before stating Lemma 3.

##### Definition 1

(Local minimum). A vector ${\vec {x}}^{* }\in X$ is a local minimum of the objective function *f* over the feasible set *X* if there exists an *ϵ* > 0 such that $f({\vec {x}}^{* })\le f(\vec {x})$ for all $\vec {x}\in X$ where $| | \vec {x}-{\vec {x}}^{* }| | < \epsilon$.

##### Definition 2

(Active constraints). The set of active inequality constraints $A(\vec {x})$ at a point $\vec {x}\in X$ is the set of indices of the inequality constraints that are satisfied as equalities at $\vec {x}$, i.e., $A(\vec {x})=\{j| {g}_{j}(\vec {x})=0\}$.

##### Definition 3

(Regularity). A feasible vector $\vec {x}$ is regular if the gradients of all the equality constraints $\nabla {h}_{i}(\vec {x}),i=1,\ldots ,m$, and the gradients of all the active inequality constraints $\nabla {g}_{j}(\vec {x}),j\in A(\vec {x})$, are linearly independent.

##### Lemma 3

(Proposition 3.3.1 in ref. ^54^). Let ${\vec {x}}^{* }$ be a local minimum of Problem 1 where *f*, *h*_*i*_, *g*_*j*_ are continuously differentiable functions from ${{\mathbb{R}}}^{d}$ to ${\mathbb{R}}$, and assume that ${\vec {x}}^{* }$ is regular. Then, there exist unique Lagrange multiplier vectors ${\vec{\lambda }}^{* }={({\lambda }_{1}^{* },\ldots ,{\lambda }_{m}^{* })}^{T}\in {{\mathbb{R}}}^{m}$ and ${\vec{\mu }}^{* }={({\mu }_{1}^{* },\ldots ,{\mu }_{r}^{* })}^{T}\in {{\mathbb{R}}}^{r}$, such that

28 $$\left\{\begin{array}{rcl}&&{\nabla }_{x}L({\vec {x}}^{* },{\vec{\lambda }}^{* },{\vec{\mu }}^{* })=\vec{0}\\ &&{\mu }_{j}^{* }\ge 0,\quad j=1,\ldots ,r,\\ &&{\mu }_{j}^{* }=0,\quad \forall \,j\,\notin\, A({\vec {x}}^{* }),\end{array}\right.$$

where $A({\vec {x}}^{* })$ is the set of active constraints at ${\vec {x}}^{* }$.

##### Proof of Proposition 3

We will first translate our optimization problem into the form in Problem 1, where we have $f(\vec {x})=-\mathop{\sum }\nolimits_{i = 1}^{{d}^{2}}{x}_{i}^{4}$, ${h}_{j+1}(\vec {x})=\mathop{\sum }\nolimits_{i = jd+1}^{jd+d}{x}_{i}^{2}-1$, ${g}_{2jd+k+1}(\vec {x})={x}_{jd+k+1}-\sqrt{{c}_{\max }}$, and ${g}_{(2j+1)d+k+1}(\vec {x})=\sqrt{{c}_{\min }}-{x}_{jd+k+1}$ for *j*, *k* = 0, …, *d* − 1, where $0\le {c}_{\min }\le {c}_{\max }\le 1$. This is in fact a sum of *d* independent optimization problems of identical form, which simplifies our whole optimization problem to:

29 $$\begin{array}{lll}{\text{minimize}} \quad \,\,f(\vec {x})&=&-d\mathop{\sum }\limits_{i=1}^{d}{x}_{i}^{4}\\ \,{\rm{subject}}\, {\rm{to}}\quad h(\vec {x})&=&\mathop{\sum }\limits_{i=1}^{d}{x}_{i}^{2}-1=0,\\\qquad\qquad\quad {g}_{k}(\vec {x})&=&{x}_{k}-\sqrt{{c}_{\max }}\le 0,\\\quad\qquad\quad \,{g}_{d+k}(\vec {x})&=&\sqrt{{c}_{\min }}-{x}_{k}\le 0,\,k=1,\ldots ,d,\end{array}$$

where we redefine the objective function $f(\vec {x})$. The associated Lagrangian is given by

30 $$\begin{array}{lll}L(\vec {x},\lambda ,\vec{\mu })\,=\,\mathop{\sum }\limits_{i=1}^{d}\left[-d{x}_{i}^{4}+\lambda {x}_{i}^{2}+({\mu }_{i}-{\mu }_{i+d}){x}_{i}\right] \\ \qquad\qquad\,-\lambda -\mathop{\sum }\limits_{i=1}^{d}(\sqrt{{c}_{\max }}{\mu }_{i}-\sqrt{{c}_{\min }}{\mu }_{i+d}),\end{array}$$

where $\lambda ,{\mu }_{j}\in {\mathbb{R}}$. Let us determine which $\vec {x}$ is regular by evaluating the following gradients:

31 $$\nabla h(\vec {x})=\sum _{i}2{x}_{i}| i\left.\right\rangle ,\,\nabla {g}_{i}(\vec {x})=| i\left.\right\rangle ,\,\nabla {g}_{i+d}(\vec {x})=-| i\left.\right\rangle .$$

If ${g}_{i}(\vec {x})\le 0$ is active (i.e., ${x}_{i}=\sqrt{{c}_{\max }}$), then ${g}_{i+d}(\vec {x})\le 0$ for the same *i* must be inactive unless ${c}_{\min }={c}_{\max }$, and vice versa. Note that the only feasible solution of the case when ${c}_{\min }={c}_{\max }$ are non-regular since both ${g}_{i}(\vec {x})\le 0$ and ${g}_{i+d}(\vec {x})\le 0$ are active for all *i*, so $\nabla h(\vec {x}),\nabla {g}_{i}(\vec {x})$, and $\nabla {g}_{i+d}(\vec {x})$ for *i* = 1, …, *d* are linearly dependent. Also, if $\sqrt{{c}_{\min }} < {x}_{i} < \sqrt{{c}_{\max }}$, then both ${g}_{i}(\vec {x})\le 0$ and ${g}_{i+d}(\vec {x})\le 0$ are inactive. Hence, for ${\vec {x}}^{* }$ to be regular, the following must hold:

(i) At least one component ${x}_{i}^{* }$ must satisfy $\sqrt{{c}_{\min }} < {x}_{i}^{* } < \sqrt{{c}_{\max }}$ so that both ${g}_{i}({\vec {x}}^{* })\le 0$ and ${g}_{i+d}({\vec {x}}^{* })\le 0$ are inactive.
(ii) In case ${c}_{\min }=0$, if at least one component ${x}_{i}^{* }=\sqrt{{c}_{\min }}=0$ so that the *i*-th component of $\nabla h({\vec {x}}^{* })$ is 0 while $\nabla {g}_{i+d}({\vec {x}}^{* })=-| i\left.\right\rangle$, then condition (i) is not necessary. If ${x}_{i}^{* } > 0\,\forall \,i$, condition (i) must hold.

Since the functions *f*, *h*, *g*_*i*_, *g*_*i*+*d*_ for all *i* = 1, …, *d* are continuously differentiable, all regular local minima of Problem (29) must satisfy Lemma 3. Let us evaluate

32 $${\nabla }_{x}L=\mathop{\sum }\limits_{i=1}^{d} \left(-4d{x}_{i}^{3}+2\lambda {x}_{i}+{\mu }_{i}-{\mu }_{i+d}\right)| i\left.\right\rangle ,$$

and consider the case where the *i*-th component of a regular local minimum ${\vec {x}}^{* }$ satisfies $\sqrt{{c}_{\min }} < {x}_{i}^{* } < \sqrt{{c}_{\max }}$. By Lemma 3, ${\mu }_{i}^{* }={\mu }_{i+d}^{* }=0$, implying that $-4d{({x}_{i}^{* })}^{3}+2{\lambda }^{* }{x}_{i}^{* }=0$. Hence,

33 $${\lambda }^{* }=2d{({x}_{i}^{* })}^{2}.$$

Since λ^*^ is a parameter independent of the index *i*, we can constrain all components of ${\vec {x}}^{* }$ that lie within the interval, $(\sqrt{{c}_{\min }},\sqrt{{c}_{\max }})$, with this common parameter. According to eq. (33), ${x}_{i}^{* }={x}_{j}^{* }=\sqrt{{\lambda }^{* }/(2d)}$ for all *i*, *j* such that $\sqrt{{c}_{\min }} < {x}_{i}^{* },{x}_{j}^{* } < \sqrt{{c}_{\max }}$. Hence, for any regular local minimum ${\vec {x}}^{* }$ of the optimization problem (29), each component ${x}_{i}^{* }$ can only take one of the three possible values: $\sqrt{{c}_{\min }},\sqrt{\chi },\sqrt{{c}_{\max }}$, where $\chi \in ({c}_{\min },{c}_{\max })$.

Therefore, we can translate our optimization problem of (29) into a much simplified form:

34 $$\begin{array}{ll}\,\text{maximize}\;\;d[L{c}_{\max }^{2}+\overline{L}{\chi }^{2}+(d-L-\overline{L}){c}_{\min }^{2}]\\ \;\text{subject}\;\text{to}\;\;L{c}_{\max }+\overline{L}\chi +(d-L-\overline{L}){c}_{\min }=1,\\\qquad\qquad\quad \,0\le L+\overline{L}\le d,\quad L,\overline{L}\in {\mathbb{N}},\\\qquad\qquad\quad \;0\le {c}_{\min } < \chi < {c}_{\max }\le 1,\end{array}$$

where we converted our problem back to a maximization problem. Clearly, the optimal solution can be obtained by maximizing *L* while satisfying all constraints, including eq. (34) which can be rearranged into:

35 $$L=\frac{1-{c}_{\min }d}{{c}_{\max }-{c}_{\min }}-\overline{L}\,\frac{\chi -{c}_{\min }}{{c}_{\max }-{c}_{\min }}.$$

Since the last fraction lies within the interval (0, 1), the maximum value allowed for *L* is $\lfloor \frac{1-{c}_{\min }d}{{c}_{\max }-{c}_{\min }}\rfloor$, leaving either $\overline{L}=0$ if $\frac{1-{c}_{\min }d}{{c}_{\max }-{c}_{\min }}\in {\mathbb{N}}$, or $\overline{L}=1$ with $\chi =1-L{c}_{\max }-(d-L-1){c}_{\min }$ otherwise.

Next, we consider the remaining cases: (1) regular local minima ${\vec {x}}^{* }$ with no components satisfying $\sqrt{{c}_{\min }} < {x}_{i}^{* } < \sqrt{{c}_{\max }}$ when ${c}_{\min }=0$, and (2) non-regular points $\vec {x}$ with each component ${x}_{i}\in \{\sqrt{{c}_{\min }} > 0,\sqrt{{c}_{\max }}\}$ [see conditions (i) and (ii)]. Note that the case where ${c}_{\min }={c}_{\max }$ is included here. Similar to the previous regular cases, we can simplify our optimization problem to:

36 $$\begin{array}{ll}\,{\text{maximize}}\;\; {d}[L{c}_{\max }^{2}+(d-L){c}_{\min }^{2}]\\ \,{\rm{subject}}\, {\rm{to}}\;\;L{c}_{\max }+(d-L){c}_{\min }=1,\\ \,\qquad\qquad\;\;\, 0\le L\le d,\,L\in {\mathbb{N}},\,0\le {c}_{\min }\le {c}_{\max }\le 1.\end{array}$$

If ${c}_{\min } < {c}_{\max }$, the problem has a feasible optimum only if $L=\frac{1-{c}_{\min }d}{{c}_{\max }-{c}_{\min }}\in {\mathbb{N}}$. If ${c}_{\min }={c}_{\max }$, the problem has a feasible solution only if ${c}_{\min }={c}_{\max }=\frac{1}{d}$.

Finally, since the global optimum to our initial Problem (29) is the minimum over the set composed of all feasible points fulfilling Lemma 3 (a set containing all regular local minima) together with all irregular feasible solutions, we can conclude that the global optimum ${\vec {x}}^{* }$ satisfies: ${x}_{i}^{* }=\sqrt{{c}_{\max }}$ for *i* = 1, …, *L*, ${x}_{j}^{* }=\sqrt{{c}_{\min }}$ for *j* = *L* + 1, …, *d* − 1, and ${x}_{d}^{* }=\sqrt{1-L{c}_{\max }-(d-L-1){c}_{\min }}$, where

37 $$L=\left\{\begin{array}{ll}\left\lfloor \frac{1-{c}_{\min }d}{{c}_{\max }-{c}_{\min }}\right\rfloor \quad &\,\text{if}\,\,\,{c}_{\max } > {c}_{\min },\\ d\quad &\,\text{if}\,\,\,{c}_{\max }={c}_{\min }.\end{array}\right.$$

□

## Supplementary information

Supplementary information

## Data Availability

Data sharing is not applicable to this article as no datasets were generated or analysed during the current study.
