# Supplementary material for: High-dimensional entanglement witnessed by correlations in arbitrary bases
Source: npj Quantum Inf. 2025 Mar 19;11(1):50. doi: 10.1038/s41534-025-00990-6 (PMC11922771; doi:10.1038/s41534-025-00990-6)
Supplement: Supplementary file 1 — Supplementary information [file 41534_2025_990_MOESM1_ESM.pdf]

Supplementary Information for "High-dimensional entanglement witnessed by correlations in arbitrary bases"

Nicky Kai Hong Li,[1,2,3,*] Marcus Huber,[1,3] and Nicolai Friis[1,3]

[1] *Atominstitut, Technische Universität Wien, Stadionallee 2, 1020 Vienna, Austria*
[2] *Vienna Center for Quantum Science and Technology, TU Wien, 1020 Vienna, Austria*
[3] *Institute for Quantum Optics and Quantum Information (IQOQI), Austrian Academy of Sciences, Boltzmanngasse 3, 1090 Vienna, Austria*

(Dated: February 27, 2025)

This Supplementary Information, where we present additional details and explicit calculations that support our results, is structured as follows: in Sec. S.I, we provide additional analyses on the performance of our Schmidt-number witness and lower bound of the entanglement fidelity (or the singlet fraction [1, 2]) when applied to isotropic states and noisy purified thermal states. In the same section, we also compare the noise tolerance of our witness with that of Ref. [3] when applied to the two types of states, but the full details of applying Ref. [3]'s witness are deferred to Sec. S.II. In Sec. S.III, we use Lévy's lemma to prove that any two bases from a set of orthonormal bases which are chosen uniformly at random have exponentially decreasing probability of being biased as the dimension increases. In Sec. S.IV, we prove Ineq. (13) which relates the maximal number of orthonormal bases to the function $\lambda(\mathcal{C})$ defined in Theorem 1. In Sec. S.V, we present the proof of Lemma 2 which states a new construction of three MUBs that has a simple analytic form and works for any dimension $d \in \mathbb{N}$. Finally, in Sec. S.VI, we explore whether using AMUBs has any advantage in witnessing the Schmidt number using our witness in dimensions where the maximum number of MUBs is unknown.

S.I. EXAMPLES FOR VIOLATION OF OUR SCHMIDT-NUMBER WITNESS

In this section, we provide the full details of how we certify the Schmidt number and lower bound the entanglement fidelity of isotropic states in Sec. S.I.1 and the purified thermal states mixed with white noise in Sec. S.I.2.

S.I.1. Isotropic states

Let us first recall some properties of an isotropic state, i.e., a qudit Bell state mixed with a certain amount of white noise: $\rho_{AB}^{\text{iso}} = (1-p)\,|\Phi_d^+\rangle\!\langle\Phi_d^+| + \frac{p}{d^2}\mathbb{1}_{d^2}$. It has been shown that its Schmidt number is exactly $k+1$ if and only if the white-noise ratio satisfies $\frac{d(d-k-1)}{d^2-1} \leq p < 1 - \frac{kd-1}{d^2-1} = \frac{d(d-k)}{d^2-1} =: p_{\text{iso}}^{(k)}$ [4]. As mentioned in the main text,

$$\mathcal{S}_d^{(m)}(\rho_{AB}^{\text{iso}}) = p\frac{m}{d} + (1-p)m \tag{S.1}$$

and for it to exceed the bound $\mathcal{B}_k$ in Theorem 1, the white-noise ratio must satisfy

$$p < \frac{(m-\mathcal{T}(\mathcal{C}))(d-k)}{m(d-1)} =: p_{c,m}^{(k)}. \tag{S.2}$$

In the case when $d+1$ MUBs exist and $m = d+1$, it holds that $p_{c,m}^{(k)} = p_{\text{iso}}^{(k)}$ for all k.

To illustrate that our lower bound for entanglement fidelity [Ineq. (5) in Theorem 1] is valid, we will show that the inequality holds for isotropic states. As the entanglement fidelity of ρ_{AB}^{iso} is given by

$$\mathcal{F}(\rho_{AB}^{\text{iso}}) = 1 - p + \frac{p}{d^2}, \tag{S.3}$$

it is easy to see that Ineq. (5) holds whenever $m \leq \mathcal{T}(\mathcal{C})(d+1)$. When $\mathcal{T}(\mathcal{C}) = m$, this inequality is trivially satisfied. When $\mathcal{T}(\mathcal{C}) = \lambda(\mathcal{C}) < m$, the upper bound of orthonormal bases, $\overline{m}_d$, defined in Corollary S.1 satisfies $\overline{m}_d < \lambda(\mathcal{C})(d+1)$ for all $d \geq 2$. Therefore, we have $m \leq \overline{m}_d < \mathcal{T}(\mathcal{C})(d+1)$, implying that $\mathcal{F}(\rho_{AB}^{\text{iso}}) \geq \mathcal{F}_m$.

An important aspect that we should consider is how the performance of our Schmidt-number witness scales with the local dimension. With m "worst-case" measurement bases parametrized by $c_{\min}$ [5], which corresponds to the same measurement settings of the main text's example, it must hold that

$$c_{\min} > \frac{3d - 1 - \sqrt{(d+1)^2 + \frac{2(d-1)}{m(m-1)}\left\{\left[1+2m\left(\frac{p(d-1)}{d-k}-1\right)\right]^2 - 1\right\}}}{2d(d-1)} \tag{S.4}$$

for our method to witness the Schmidt number of the isotropic state ρ^{iso} with white-noise ratio p to be at least $k+1$. In Fig. S.1, we plot the suprema of the dimension-rescaled bases bias, $d\epsilon_{\min} \coloneqq 1 - dc_{\min} \in [0,1]$, below which our method using two measurement bases can witness the Schmidt number of ρ^{iso} with $p = 0.005$ to be at least $k+1$, against the local dimension d. As a benchmark, we compare these with the suprema of bases-bias tolerance for witnessing Schmidt numbers $2 \leq k+1 \leq d$ in the maximally entangled state (i.e., $p = 0$), which turns out to be independent of k as one can see

* kai.li@tuwien.ac.at

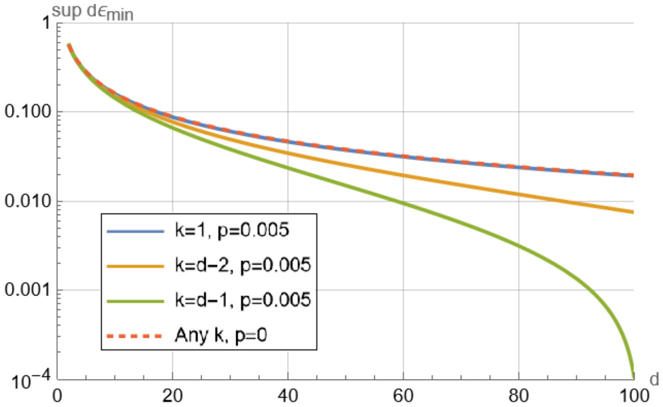

FIG. S.1. The suprema of the dimension-rescaled bases bias, $d\epsilon_{\min} \coloneqq 1 - dc_{\min} \in [0,1]$, below which our method using 2 measurement bases can witness the Schmidt number of the isotropic state with $p = 0.005$ to be at least $k+1$, are plotted on a log scale against the local dimension d. These are compared with the supremum of bases-bias tolerance for witnessing any Schmidt number $2 \le k+1 \le d$ in the maximally entangled state (i.e., $p = 0$), represented by the dotted curve.

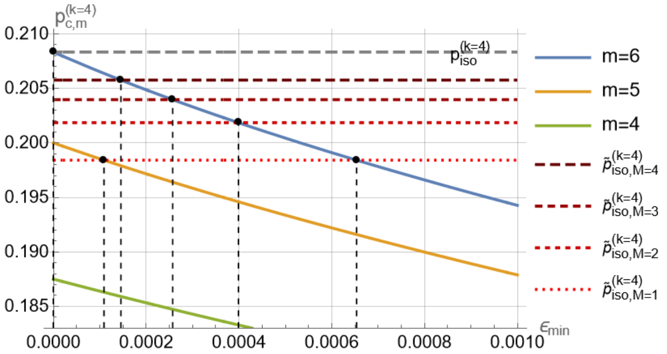

FIG. S.2. The maximal white-noise ratio, $p_{c,m}^{(k=4)}$, below which our witness can certify $\rho_{AB}^{\rm iso}$'s Schmidt number to be $k+1=5$ for different number of measurement bases m are plotted against the bases-bias parameter, $\epsilon_{\min} \coloneqq 1/d - c_{\min}$. For $m=5$ and 6, they coincide with the white-noise thresholds of Ref. [3], $\tilde{p}_{{\rm iso},M}^{(k=4)}$, at different values of $\epsilon_{\min}$ (labelled by black dots), where M is the number of "tilted" measurement bases used. For $m \le 4$, $p_{c,m}^{(k=4)}$ are strictly smaller than $\tilde{p}_{{\rm iso},M}^{(k=4)}$ for all $1 \le M \le d$. The white-noise threshold $\tilde{p}_{{\rm iso},M=5}^{(k=4)}$, which is not shown here, coincides with the white-noise ratio $p_{\rm iso}^{(k=4)}$ for $\rho_{AB}^{\rm iso}$ to have the maximum Schmidt number ($d=5$).

from Ineq. (S.4) when $p = 0$. At least for the worst-case bases choice, this figure and Ineq. (S.4) suggest that our witness tolerates less and less bias in the measurement bases until it cannot certify any Schmidt number of isotropic states as the local dimension increases. We suspect that this is a general feature of our witness as the bounds $\mathcal{B}_k$ ($\overline{\mathcal{B}_k}$) in Theorem 1 (Lemma 1) have a bases-bias-dependent term that grows at least in $\mathcal{O}(\sqrt{d})$, reducing the tolerance to bases bias in larger dimensions.

Next, we will compare the performance of our witness with the one proposed in Ref. [3] using the example of isotropic states $\rho_{AB}^{\rm iso}$ [6]. The main idea behind the Schmidt-number witness proposed in Ref. [3] is summarized in Sec. S.II.1. The analytic expressions of the entanglement fidelity lower bound and the white-noise tolerance for each Schmidt number of the isotropic states $\rho_{AB}^{\rm iso}$ are derived in Sec. S.II.2.

In Fig. S.2, we fix the local dimension $d=5$ and plot the maximal white-noise ratios, $p_{c,m}^{(k=4)}$ and $\tilde{p}_{{\rm iso},M}^{(k=4)}$, below which our witness and the witness in Ref. [3] can certify $\rho_{AB}^{\rm iso}$'s Schmidt number to be $k+1=5$, respectively. Note that m denotes the total number of orthonormal measurement bases used in our setting, whereas M denotes the total number of "tilted" (potentially non-orthogonal) bases used in Ref. [3], which does not count the computational basis that they also need to measure in (see Sec. S.II.1). For isotropic states, the "tilted" bases are orthonormal and form a set of MUBs (see Sec. S.II.2). Hence, we can make the association, $m = M+1$, for an intuitive comparison between Ref. [3]'s and our methods. As the minimum bases overlap $c_{\min}$ reduces from the maximum value $\frac{1}{d} = 0.2$ (i.e., $\epsilon_{\min} = \frac{1}{d} - c_{\min}$ increases from 0), our white-noise threshold $p_{c,m}^{(k=4)}$ decreases. For $m=6$, $p_{c,m}^{(k=4)}$ coincides with Ref. [3]'s thresholds $\tilde{p}_{{\rm iso},M}^{(k=4)}$ at $\epsilon_{\min} \approx 0.00065$ ($M=1$), 0.00040 ($M=2$), 0.00026 ($M=3$), 0.00015 ($M=4$), and precisely 0 ($M=5$). For $m=5$, $p_{c,m}^{(k=4)}$ only coincides with $\tilde{p}_{{\rm iso},M=1}^{(k=4)}$ at $\epsilon_{\min} \approx 0.00011$ and not for any $M \ge 2$. Furthermore, $p_{c,m}^{(k=4)}$ does not coincide with $\tilde{p}_{{\rm iso},M}^{(k=4)}$ for all $m \le 4$, $1 \le M \le 5$ and $\epsilon_{\min} \in [0, 0.2]$.

We also compare our entanglement-fidelity lower bounds of the isotropic states $\rho^{\rm iso}$ with the bounds from Ref. [3]. In Fig. S.3 we plot the square root of the relative differences between the actual entanglement fidelity of $\rho_{AB}^{\rm iso}$ and our fidelity lower bounds, $\sqrt{\Delta\overline{\mathcal{F}}_m^{\rm iso}} \coloneqq \sqrt{1 - \overline{\mathcal{F}}_m/\mathcal{F}(\rho_{AB}^{\rm iso})}$ [7], associated to measuring in m orthonormal bases with different minimum overlaps $c_{\min}$, and the equivalent square-root relative fidelity differences from Ref. [3], $\sqrt{\Delta\tilde{\mathcal{F}}^{(M)}} \coloneqq \sqrt{1 - \tilde{\mathcal{F}}^{(M)}/\mathcal{F}(\rho_{AB}^{\rm iso})}$, for measuring in M "tilted" bases (which are MUBs in this example), against different white-noise ratios p.

Although Figs. S.2 and S.3 suggest that our Schmidt-number witness and entanglement fidelity lower bound require more measurement bases than those in Ref. [3] to achieve comparable performance, one should keep in mind that Ref. [3] requires the measurement bases to be exact MUBs in this example or, in general, to be the "tilted" bases with strict specifications of the relative phases. On the contrary, our method does not require the measurement bases to satisfy any relative phase relationship. In particular, we can still witness Schmidt number and lower bound the entanglement fidelity when measurements in MUBs are not experimentally feasible.

Furthermore, Figs. S.2 and S.3 may suggest that our method only tolerates very small bases bias. However,

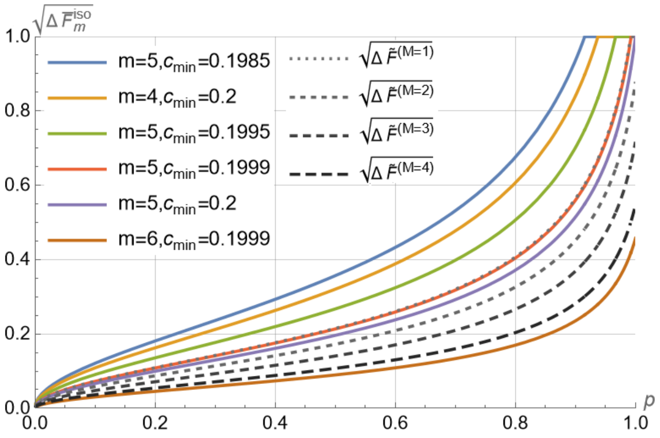

FIG. S.3. The square-root relative differences between the actual entanglement fidelity of $\rho_{AB}^{\rm iso}$ and our fidelity lower bounds, $\sqrt{\Delta\overline{\mathcal{F}}_m^{\rm iso}} \coloneqq \sqrt{1-\overline{\mathcal{F}}_m/\mathcal{F}(\rho_{AB}^{\rm iso})}$, associated to measuring in m bases with different minimum overlaps $c_{\rm min}$ are plotted against different white-noise ratios p. They are compared against the square-root relative differences between the actual entanglement fidelity and the fidelity lower bounds in Ref. [3], $\sqrt{\Delta\tilde{\mathcal{F}}^{(M)}} \coloneqq \sqrt{1-\tilde{\mathcal{F}}^{(M)}/\mathcal{F}(\rho_{AB}^{\rm iso})}$, for measuring in M "tilted" bases (which are coincidentally MUBs in this example). Both $\Delta\overline{\mathcal{F}}_{d+1}^{\rm iso}$ at $c_{\rm min} = 1/d$ and $\Delta\tilde{\mathcal{F}}^{(M=d)}$ for $\rho_{AB}^{\rm iso}$ are zero, and they are omitted from the plot.

one should recall that we picked the worst-case bases choice for any given $c_{\rm min}$ [5]. In practice, measuring in any other bases associated with a fixed $c_{\rm min}$ will give better noise tolerance (i.e., witnessing higher Schmidt number or providing higher fidelity lower bound).

S.I.2. Purified thermal states mixed with white noise

In this example, we want to investigate (1) how the evenness of the eigenvalues of the one-party reduced density matrix ρ_A affects the performance of our witness when applied to ρ_{AB}, and (2) whether measuring in an additional basis that is not unbiased with respect to the other measurement bases which are MUBs improve the performance of our witness.

We consider the purified thermal states mixed with white noise parametrized by $p \in [0,1]$ and $\beta \in [0,\infty)$:

$$\rho_{\rm th}(p,\beta) = (1-p)\,|\psi_{\rm th}(\beta)\rangle\langle\psi_{\rm th}(\beta)| + \frac{p}{d^2}\mathbb{1}_{d^2}, \qquad \text{(S.5a)}$$

$$|\psi_{\rm th}(\beta)\rangle = \frac{1}{\sqrt{\mathcal{Z}}}\sum_{n=0}^{d-1} e^{-\frac{\beta n}{2}}\,|n\rangle\otimes|n\rangle\,, \qquad \text{(S.5b)}$$

where $\mathcal{Z} = \sum_{n=0}^{d-1} e^{-\beta n}$. The reduced density matrix is

$$\rho_{{\rm th},A}(p,\beta) = \sum_{n=0}^{d-1}\left[(1-p)\frac{e^{-\beta n}}{\mathcal{Z}} + \frac{p}{d}\right]|n\rangle\langle n|\,, \qquad \text{(S.6)}$$

which has uneven eigenvalues for $\beta > 0$. These states are interesting since they are known to be not optimal for the Schmidt-number witness in Ref. [3]. Also, by changing just one parameter β, $|\psi_{\rm th}(\beta)\rangle$ goes from being the maximally entangled state to an entangled state with d positive uneven Schmidt coefficients, and finally to the separable state $|0\rangle\otimes|0\rangle$ as β goes from 0 to ∞.

For simplicity, we consider only odd-prime dimensions d in this example. Suppose that there are three local measurement bases available in our experiment: $\{|e_i^1\rangle = |i\rangle\}_{i=0}^{d-1}$, $\{|e_j^2\rangle = \frac{1}{\sqrt{d}}\sum_{n=0}^{d-1}\omega^{jn}\,|n\rangle\}_{j=0}^{d-1}$ and $\{|e_j^3\rangle\}_{j=0}^{d-1}$ with

$$|e_j^3\rangle = Z_\alpha(\theta)\sum_{k=0}^{d-1}\frac{\omega^{jk+k^2}}{\sqrt{d}}\,|k\rangle \qquad \text{(S.7)}$$

where $\omega = e^{i\frac{2\pi}{d}}$, $Z_\alpha(\theta) = \sum_{k=0}^{d-1} e^{i\delta_{\alpha,k}\theta}\,|k\rangle\langle k|$ with $\alpha \in [d] \coloneqq \{0,\ldots,d-1\}$ and $\theta \in [0,2\pi)$, and $\delta_{\alpha,k}$ is the Kronecker delta. The first two bases and bases 1 and 3 are both pairwise mutually unbiased, but the three bases together do not form a set of MUBs except when $\theta = 0$ [8]. Therefore, one can interpret them as an imperfect implementation of three MUBs with the third basis subject to a phase drift $Z_\alpha(\theta)$ relative to the second basis.

Let us first evaluate the expectation value of our witness operator [see Eq. (1) in the main text]

$$\mathcal{S}_d^{(m)}(\rho_{\rm th}) = (1-p)\sum_{z=1}^{m}\sum_{a=0}^{d-1}|\langle e_a^z,\tilde{e}_a^{z*}|\psi_{\rm th}(\beta)\rangle|^2 + \frac{mp}{d}. \quad \text{(S.8)}$$

For each matching pair of local measurement bases, we have

$$\sum_{a=0}^{d-1}|\langle e_a^1,\tilde{e}_a^{1*}|\psi_{\rm th}(\beta)\rangle|^2 = 1, \qquad \text{(S.9a)}$$

$$\sum_{a=0}^{d-1}|\langle e_a^2,\tilde{e}_a^{2*}|\psi_{\rm th}(\beta)\rangle|^2 = \sum_{a=0}^{d-1}|\langle e_a^3,\tilde{e}_a^{3*}|\psi_{\rm th}(\beta)\rangle|^2 \qquad \text{(S.9b)}$$
$$= \begin{cases} 1 & \text{if } \beta = 0, \\ \frac{1}{d\mathcal{Z}}\left(\frac{1-e^{-d\beta/2}}{1-e^{-\beta/2}}\right)^2 & \text{if } \beta > 0. \end{cases}$$

If we combine Eqs. (S.8)–(S.9b) and simplify the expression with $\mathcal{Z} = \sum_{n=0}^{d-1} e^{-\beta n} = \frac{1-e^{-d\beta}}{1-e^{-\beta}}$ for all $\beta > 0$, $\tanh(x) = \frac{1-e^{-2x}}{1+e^{-2x}}$, and $\frac{1-e^{-x}}{1-e^{-x/2}} = 1 + e^{-\frac{x}{2}}$, we get

$$\mathcal{S}_d^{(m)}(\rho_{\rm th}) = (1-p)\tau_d^{(m)}(\beta) + \frac{mp}{d}, \qquad \text{(S.10a)}$$

$$\tau_d^{(m)}(\beta) \coloneqq \begin{cases} m & \text{if } \beta = 0, \\ 1 + \frac{(m-1)\tanh(d\beta/4)}{d\tanh(\beta/4)} & \text{if } \beta > 0, \end{cases} \qquad \text{(S.10b)}$$

depending on whether we use only the first two bases ($m = 2$) or all three bases ($m = 3$).

Our next step is to determine the upper bound of $\mathcal{S}_d^{(m)}$ for any bipartite state with Schmidt number at most k.

This requires us to calculate all the bases overlaps:

$$|\langle e_a^1|e_{a'}^z\rangle|^2 = \frac{1}{d}\ (= c_{\min}^{1,z} = c_{\max}^{1,z}), \quad \forall\ z \in \{2,3\}, \tag{S.11a}$$

$$\langle e_a^2|e_{a'}^3\rangle = \frac{1}{d}\left[\omega^{(a'-a)\alpha+\alpha^2}(e^{i\theta}-1) + \sum_{k=0}^{d-1}\omega^{(a'-a)k+k^2}\right], \tag{S.11b}$$

$$|\langle e_a^2|e_{a'}^3\rangle|^2 = \frac{1}{d^2}\left\{\left|\sum_{k=0}^{d-1}\omega^{(a'-a)k+k^2}\right|^2 + |e^{i\theta}-1|^2 + 2\mathrm{Re}\left[\omega^{(a'-a)\alpha+\alpha^2}(e^{i\theta}-1)\sum_{k=0}^{d-1}\omega^{(a'-a)k+k^2}\right]\right\} \tag{S.11c}$$

for all $a, a' \in [d]$. With $-|y| \leq \mathrm{Re}(y) \leq |y|$ for all $y \in \mathbb{C}$ and a fact regarding quadratic Gauss sums [8], i.e.,

$$\left|\sum_{k=0}^{d-1}\omega^{ak+k^2}\right| = \sqrt{d} \quad \forall\ a \in \mathbb{Z}\ \&\ \text{odd prime } d, \tag{S.12}$$

we obtain the upper and lower bounds for $|\langle e_a^2|e_{a'}^3\rangle|^2$ as

$$c_\pm^{2,3} = \frac{1}{d^2}\left(\sqrt{d} \pm 2\,|\sin(\theta/2)|\right)^2, \tag{S.13}$$

such that $c_{\min}^{2,3} = c_-^{2,3} \leq |\langle e_a^2|e_{a'}^3\rangle|^2 \leq c_+^{2,3} = c_{\max}^{2,3}$. Then, applying the formula for $\overline{\mathcal{B}}_k$ in Lemma 1, we obtain for $m = 2$ or 3, $\overline{\mathcal{T}}(\mathcal{C}) \coloneqq \min\{\overline{\lambda}(\mathcal{C}), m\}$ with

$$\overline{\lambda}(\mathcal{C}) = \frac{1}{2}\left(1 + \sqrt{1 + 4d\delta_{m,3}\overline{G}^{\,2,3}}\right), \tag{S.14}$$

with the shorthand notation $\overline{G}^{\,z,z'} \coloneqq \overline{G}(c_{\max}^{z,z'}, c_{\min}^{z,z'})$ and the fact that $\overline{G}^{\,1,z} = \overline{G}^{\,z,1} = 0$ and $\overline{G}^{\,2,3} = \overline{G}^{\,3,2} = 1-(d+1)c_{\min}^{2,3} + \Omega^{2,3}$ as defined in Lemma 1.

In order to witness $\rho_{\text{th}}(p,\beta)$'s Schmidt number is at least $k+1$, the relationship $\mathcal{S}_d^{(m)}(\rho_{\text{th}}) > \overline{\mathcal{B}}_k$ must be satisfied, which after some manipulation gives

$$p < \frac{\tau_d^{(m)}(\beta)d - km - (d-k)\overline{\mathcal{T}}(\mathcal{C})}{\tau_d^{(m)}(\beta)d - m} =: p_{\text{th},m}^{(k)}(\beta,\theta) \tag{S.15}$$

if $\tau_d^{(m)}(\beta) > \frac{m}{d}$ which holds for all $\beta \in [0,\infty)$ according to the definition in Eq. (S.10b). We plot $p_{\text{th},m}^{(k)}(\beta,\theta)$, the threshold of white-noise ratio for detecting $\rho_{\text{th}}(p,\beta)$'s Schmidt number larger than k against θ with $\beta = 0.5$ and against β with $\theta = 0.05$ for $d = 5$, $m = 2, 3$ and $k = 1$ to 4 in Figs. S.4 and S.5, respectively. Both figures suggest that measuring in an additional basis that is close to forming a set of MUBs with the already measured bases can improve the noise tolerance of our witness. On the other hand, an additional measurement basis that is far from being mutually unbiased with the other bases could adversely affect the performance of our witness due to a significant increase in the upper bound $\mathcal{B}_k$ ($\overline{\mathcal{B}}_k$) in Theorem 1 (Lemma 1). We summarize this observation in Remark 1 which already appeared in the main article.

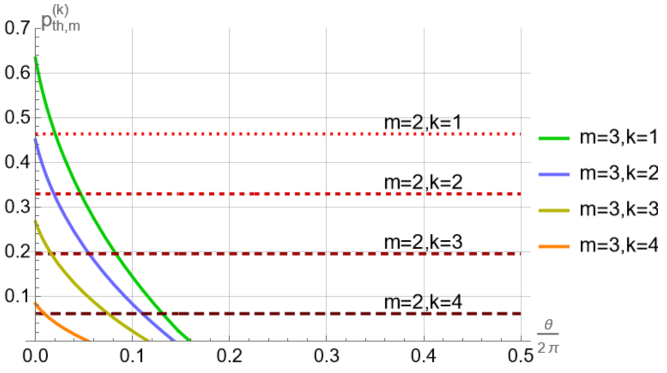

FIG. S.4. The white-noise threshold for detecting $\rho_{\text{th}}(p,\beta)$'s Schmidt number to be larger than k, $p_{\text{th},m}^{(k)}(\beta,\theta)$, plotted against the third basis' phase-drift parameter θ for $\beta = 0.5$, $d = 5$, $m = 2, 3$ and $k = 1$ to 4. Since $p_{\text{th},m}^{(k)}(\beta,\theta) = p_{\text{th},m}^{(k)}(\beta, 2\pi - \theta)$ [9], we omit the plot for $\pi \leq \theta \leq 2\pi$. The threshold for measuring in all three bases $p_{\text{th},m=3}^{(k)}(\beta,\theta)$ decreases as θ increases and eventually gets below $p_{\text{th},m=2}^{(k)}(\beta,\theta)$, the threshold for measuring only in the first two bases for all k.

Remark 1. There exist scenarios where an additional measurement basis improves the noise tolerance of our Schmidt-number witness. However, the opposite case can also happen depending on the choice of the bases. Therefore, in order to witness the highest Schmidt number of a state, one should apply the witness inequality in Theorem 1 or Lemma 1 to all subsets of the total set of m' available measurement bases and find the largest k such that $\mathcal{S}_d^{(m)}(\rho) > \mathcal{B}_k$ or $\overline{\mathcal{B}}_k$ when evaluated over all subsets of m chosen bases for all $m \in \{2, \ldots, m'\}$.

We can also see how the additional measurement basis and the evenness of the reduced density matrix's eigenvalues affect our lower bound of the entanglement fidelity. The entanglement fidelity of $\rho_{\text{th}}(p,\beta)$ can be found by noticing that the absolute value of any matrix element of a unitary is upper bounded by 1 [10], which implies

$$\max_U |\langle\psi_{\text{th}}|\mathbb{1}\otimes U|\Phi^+\rangle| = \max_U \frac{1}{\sqrt{d\mathcal{Z}}}\left|\sum_{n=0}^{d-1} e^{-\frac{\beta n}{2}}\langle n|U|n\rangle\right| = \frac{1}{\sqrt{d\mathcal{Z}}}\sum_{n=0}^{d-1} e^{-\frac{\beta n}{2}}. \tag{S.16}$$

Then, applying the same methods that resulted in Eq. (S.10b) gives us $\rho_{\text{th}}(p,\beta)$'s entanglement fidelity,

$$\mathcal{F}(\rho_{\text{th}}(p,\beta)) = \begin{cases} 1 - p + \frac{p}{d^2} & \text{if } \beta = 0, \\ \frac{(1-p)\tanh(d\beta/4)}{d\tanh(\beta/4)} + \frac{p}{d^2} & \text{if } \beta > 0. \end{cases} \tag{S.17}$$

We compare our fidelity lower bound with the true value in Fig. S.6, which shows the relative difference $\Delta\overline{\mathcal{F}}_m^{\text{th}} \coloneqq 1 - \overline{\mathcal{F}}_m/\mathcal{F}(\rho_{\text{th}}(p,\beta))$ for different values of p, β and θ.

Finally, we compare the performance of our Schmidt-number witness with the one in Ref. [3]. In Fig. S.7, we

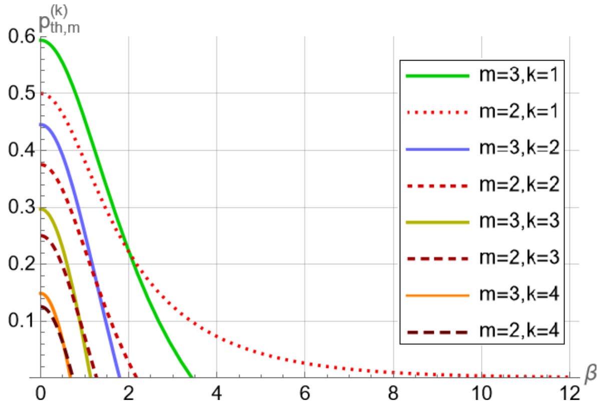

FIG. S.5. The white-noise threshold for detecting $\rho_{\rm th}(p,\beta)$'s Schmidt number being larger than k, $p^{(k)}_{{\rm th},m}(\beta,\theta)$, plotted against β for $\theta = 0.05$, $d = 5$, $m = 2,3$ and $k = 1$ to 4. All thresholds decrease as β increases, which corresponds to increasing skewness of eigenvalues of the reduced density matrix in Eq. (S.6). Due to the bias of the third basis, $p^{(k)}_{{\rm th},m=3}(\beta,0.05) - p^{(k)}_{{\rm th},m=2}(\beta,0.05)$ goes from being strictly positive to negative as β increases from 0.

plot the white-noise thresholds for detecting $\rho_{\rm th}(p,\beta)$'s Schmidt number larger than k, $p^{(k)}_{{\rm th},m}(\beta,\theta)$, against the third basis' phase-drift parameter θ for $\beta = 0.05$, $d = 5$, and $k = 1$ to 4. These are compared with the white-noise thresholds for measuring in the computational basis together with $M = 2$ "tilted" bases of Ref. [3], $\tilde{p}^{(k)}_{{\rm th},M=2}$, which are numerically computed as described in Sec. S.II.3. Similar to the example of isotropic states, Fig. S.7 suggests that the witness of Ref. [3] is more noise-tolerant than our method in certifying Schmidt numbers of $\rho_{\rm th}(p,\beta)$. However, we should not overlook that the "tilted" measurement bases are not orthogonal and require precise control in the relative phases among the bases vectors. Therefore, the choice of bases in Ref. [3] may not be applicable in general experimental settings. For instance, the natural measurement bases for certifying entanglement in certain photonic systems are the temporal and frequency bases [11], or the position and momentum bases in cold atoms [12], both of which are inherently orthogonal. It remains unclear how practical it is to realize non-orthogonal measurement bases or bases with specifically chosen relative phases in these settings. Note that full derivations of the noise tolerance of Ref. [3]'s witness can be found in Sec. S.II.3.

S.II. COMPARISON WITH THE SCHMIDT-NUMBER WITNESS IN REF. [3]

In this section, we first summarize the main idea behind the Schmidt-number witness proposed in Ref. [3]. After defining the witness, we will apply it to isotropic states and noisy purified thermal states and calculate the white-noise tolerances in both cases, which are plotted in Figs. S.2 and S.7, respectively.

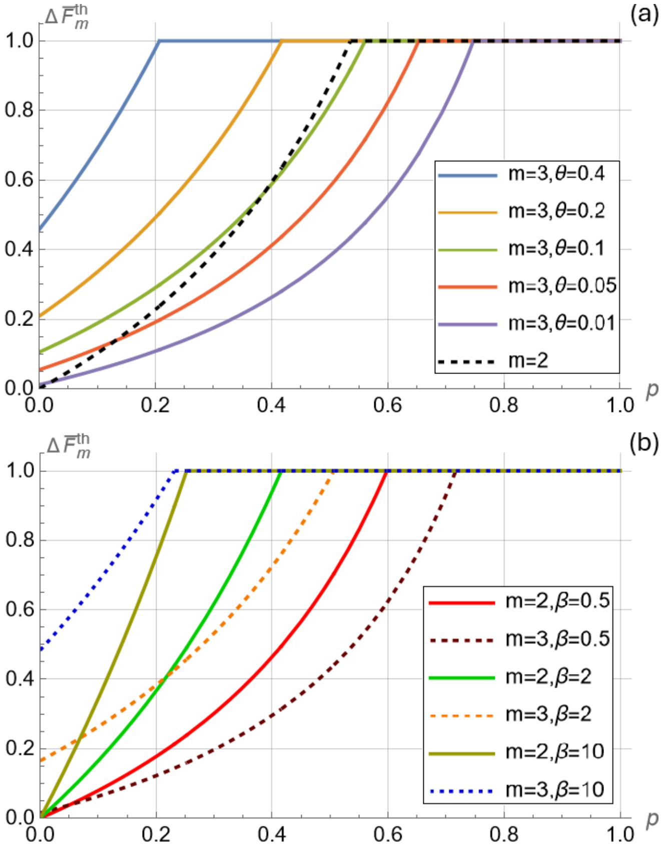

FIG. S.6. The relative differences between the actual entanglement fidelity of $\rho_{\rm th}$ and our fidelity lower bounds, $\Delta\overline{\mathcal{F}}^{\rm th}_m := 1 - \overline{\mathcal{F}}_m/\mathcal{F}(\rho_{\rm th}(p,\beta))$, associated to measuring in $m = 2,3$ bases are plotted against different white-noise ratios p, where only the third basis is parameterized by θ. In (a), β is fixed to be 1 and we plot $\Delta\overline{\mathcal{F}}^{\rm th}_m$ for different values of θ. When the third measurement basis is used, $\Delta\overline{\mathcal{F}}^{\rm th}_m$ is lower than that of using 2 MUBs for small θ and it grows beyond the relative fidelity difference of 2 MUBs as θ increases. In (b), θ is fixed to be 0.05 and we plot $\Delta\overline{\mathcal{F}}^{\rm th}_m$ for different values of β. For small p, using 3 bases is worse than using only 2 bases for the given θ. For mid-range values of p, the bounds using 3 bases are tighter for $\beta = 0.5$ and 2 but not for 10. In general, the relative fidelity difference appears to grow with β. The cusps in both plots occur when $\overline{\mathcal{F}}_m$ in Ineq. (8) in Lemma 1 transits from $\overline{\mathcal{T}}(\overline{\mathcal{C}}) < \mathcal{S}^{(m)}_d(\rho_{\rm th})$ to $\overline{\mathcal{T}}(\overline{\mathcal{C}}) \geq \mathcal{S}^{(m)}_d(\rho_{\rm th})$, where the fidelity lower bound becomes trivial (i.e., $\overline{\mathcal{F}}_m = 0$), as p increases.

S.II.1. Summary of the witness in Ref. [3]

For any bipartite state $\rho^{(\leq k)}_{AB}$ of Schmidt number at most k, $\mathcal{F}(\rho^{(\leq k)}_{AB}, |\psi\rangle)$, the fidelity between $\rho^{(\leq k)}_{AB}$ and a pure state $|\psi\rangle = \sum_{i=0}^{d-1}\lambda_i |e_i\rangle|f_i\rangle$ with known Schmidt coefficients $\{\lambda_i\}_{i=0}^{d-1}$ such that $\lambda_i \geq \lambda_j\ \forall\ i < j$ and $\sum_{i=0}^{d-1}\lambda_i^2 = 1$, where $\{|e_i\rangle\}_i, \{|f_i\rangle\}_i$ are orthonormal bases, must satisfy

$$\mathcal{F}(\rho^{(\leq k)}_{AB}, |\psi\rangle) \leq \widetilde{\mathcal{B}}_k(|\psi\rangle) := \sum_{i=0}^{k-1}\lambda_i^2. \quad \text{(S.18)}$$

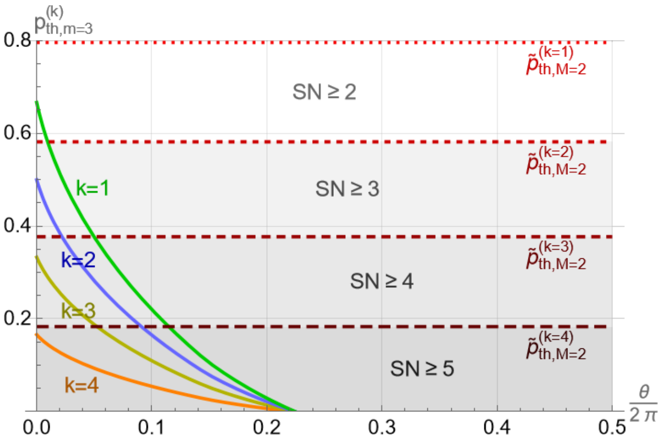

FIG. S.7. The white-noise thresholds for detecting $\rho_{\rm th}(p,\beta)$'s Schmidt number larger than k, $p^{(k)}_{{\rm th},m=3}(\beta,\theta)$, plotted against the third basis' phase-drift parameter θ for $\beta = 0.05$, $d = 5$, and $k = 1$ to 4. Since $p^{(k)}_{{\rm th},m=3}(\beta,\theta) = p^{(k)}_{{\rm th},m=3}(\beta,2\pi-\theta)$ [9], we omit the plot for $\pi \le \theta \le 2\pi$. These are compared with the white-noise thresholds for measuring in the computational basis together with $M = 2$ "tilted" bases of Ref. [3], $\tilde{p}^{(k)}_{{\rm th},M=2}$. Below the dotted lines labelled by $\tilde{p}^{(k)}_{{\rm th},M=2}$ are regions of white-noise ratios, in which $\rho_{\rm th}(p,\beta)$'s Schmidt number is certified to be at least $k+1$ by the witness of Ref. [3].

Hence, if $\mathcal{F}(\rho_{AB},|\psi\rangle) > \widetilde{\mathcal{B}}_k(|\psi\rangle)$, then we certify the Schmidt number of ρ_{AB} must be at least $k+1$.

Using as few as two measurement bases, it is possible to obtain a good estimate of $\mathcal{F}(\rho_{AB},|\psi\rangle)$ for any target state $|\psi\rangle$ in any odd-prime local dimension if the measurement bases are constructed in the way specified by Ref. [3]. For that to work, the target state $|\psi\rangle = \sum_{i=0}^{d-1}\lambda_i\,|e_i\rangle\,|f_i\rangle$ is chosen such that

$$\lambda_i = \sqrt{\frac{\langle e_i f_i|\rho_{AB}|e_i f_i\rangle}{\sum_{j=0}^{d-1}\langle e_j f_j|\rho_{AB}|e_j f_j\rangle}} \tag{S.19}$$

and $\{|e_i\rangle\}_i$ ($\{|f_i\rangle\}_i$) is the local orthonormal measurement basis of party A (B). From now on, w.l.o.g., we set $|e_i\rangle = |i\rangle$ and $|f_i\rangle = |i\rangle$ for all i.

Since calculating $\mathcal{F}(\rho_{AB},|\psi\rangle)$ (which will be abbreviated as $\mathcal{F}$ from now on) requires some knowledge of the off-diagonal entries of ρ_{AB} with respect to the local computational basis $\{|i\rangle\}$, both parties must measure in more than one basis. In Ref. [3], these extra $1 \le M \le d$ bases are chosen to be the "tilted" bases $\{|\tilde{j}_\alpha\rangle\}_{j=0}^{d-1}$ with $\alpha \in \{0,\dots,M-1\}$, which are defined by

$$|\tilde{j}_\alpha\rangle = \frac{1}{\sqrt{\sum_i \lambda_i}}\sum_{n=0}^{d-1}\omega^{jn+\alpha n^2}\sqrt{\lambda_n}\,|n\rangle\,, \tag{S.20}$$

where $\omega = e^{i\frac{2\pi}{d}}$. Given that party A (B) measures in local bases $\{i\}_{i=0}^{d-1}$ and $\{|\tilde{j}_\alpha\rangle\}_{j=0}^{d-1}$ ($\{|\tilde{j}^*_\alpha\rangle\}_{j=0}^{d-1}$), the two parties can evaluate a lower bound of the fidelity $\mathcal{F}$ given by $\tilde{F}^{(M)} \coloneqq F_1 + \tilde{F}_2^{(M)}$ [13], where

$$F_1 \coloneqq \sum_{n=0}^{d-1}\lambda_n^2\langle nn|\rho_{AB}|nn\rangle, \tag{S.21a}$$

$$\tilde{F}_2^{(M)} \coloneqq \frac{(\sum_n\lambda_n)^2}{d}\Sigma^{(M)} - \sum_{m,n=0}^{d-1}\lambda_m\lambda_n\langle mn|\rho_{AB}|mn\rangle - \sum_{\substack{m\neq m',m\neq n,\\ n\neq n',n'\neq m'}}\tilde{\gamma}^{(M)}_{mm'nn'}\sqrt{\langle m'n'|\rho_{AB}|m'n'\rangle\langle mn|\rho_{AB}|mn\rangle}\,, \tag{S.21b}$$

$$\Sigma^{(M)} \coloneqq \frac{1}{M}\sum_{\alpha=0}^{M-1}\sum_{j=0}^{d-1}\langle\tilde{j}_\alpha\tilde{j}^*_\alpha|\rho_{AB}|\tilde{j}_\alpha\tilde{j}^*_\alpha\rangle, \tag{S.21c}$$

with the quantity $\tilde{\gamma}^{(M)}_{mm'nn'}$ given by

$$\tilde{\gamma}^{(M)}_{mm'nn'} \coloneqq \frac{\tilde{\gamma}_{mm'nn'}}{M}\left|\sum_{\alpha=0}^{M-1}\omega^{\alpha(m^2-m'^2-n^2+n'^2)}\right|, \tag{S.22a}$$

$$\tilde{\gamma}_{mm'nn'} \coloneqq \begin{cases}0 & \text{if } (m-m'-n+n')\,\text{mod}\,d\neq 0,\\ \sqrt{\lambda_m\lambda_n\lambda_{m'}\lambda_{n'}} & \text{otherwise.}\end{cases} \tag{S.22b}$$

Since $\tilde{F}^{(M)} \le \mathcal{F} \le \widetilde{\mathcal{B}}_k(|\psi\rangle)$ for all ρ_{AB} with Schmidt number at most k, if $\tilde{F}^{(M)} > \widetilde{\mathcal{B}}_{k'}(|\psi\rangle)$, then ρ_{AB} must have Schmidt number at least $k'+1$.

S.II.2. Witnessing isotropic states

In this subsection, we will derive $\tilde{F}^{(M)}$ for the isotropic state $\rho^{\rm iso} = (1-p)\,|\Phi_d^+\rangle\!\langle\Phi_d^+| + \frac{p}{d^2}\mathbb{1}_{d^2}$. Since

$$\langle ij|\rho^{\rm iso}|ij\rangle = \frac{1-p}{d}\delta_{ij} + \frac{p}{d^2}, \quad \forall\, i,j\in[d], \tag{S.23}$$

the target state $|\psi\rangle$ has $\lambda_i = \frac{1}{\sqrt{d}}$ $\forall\ i$ (i.e., $|\psi\rangle = |\Phi_d^+\rangle$) according to Eq. (S.19), and the "tilted" bases together with the computational basis are MUBs in prime-power dimensions [14]. Furthermore, we have

$$F_1 = \frac{1}{d}\left(1-p+\frac{p}{d}\right), \tag{S.24a}$$

$$\Sigma^{(M)} = \frac{1}{M}\sum_{\alpha=0}^{M-1}\left(1-p+\frac{p}{d}\right) = 1-p+\frac{p}{d}, \tag{S.24b}$$

where we use the fact that $U_\alpha\otimes U_\alpha^*\,|\Phi_d^+\rangle = |\Phi_d^+\rangle$ with $U_\alpha = \sum_j|\tilde{j}_\alpha\rangle\!\langle j|$, and for the last two terms of $\tilde{F}_2^{(M)}$ we have

$$\sum_{m,n=0}^{d-1}\lambda_m\lambda_n\langle mn|\rho^{\rm iso}|mn\rangle = \frac{1}{d}{\rm Tr}(\rho^{\rm iso}) = \frac{1}{d}, \tag{S.25}$$

as well as

$$\tilde{\gamma}^{(M)}_{mm'nn'} = \begin{cases}0 & \text{if } (m-m'-n+n')\ \text{mod}\ d\neq 0,\\ \frac{1}{Md}\left|\sum_{\alpha=0}^{M-1}\omega^{\alpha(m^2-m'^2-n^2+n'^2)}\right| & \text{otherwise,}\end{cases} \tag{S.26}$$

such that

$$\sum_{\substack{m\neq m',m\neq n,\\ n\neq n',n'\neq m'}} \tilde{\gamma}^{(M)}_{mm'nn'}\sqrt{\langle m'n'|\rho^{\rm iso}|m'n'\rangle\langle mn|\rho^{\rm iso}|mn\rangle}$$

$$= \sum_{\substack{m\neq m',m\neq n,\\ n\neq n',n'\neq m'}} \tilde{\gamma}^{(M)}_{mm'nn'}\sqrt{\left(\frac{1-p}{d}\delta_{m'n'}+\frac{p}{d^2}\right)\left(\frac{1-p}{d}\delta_{mn}+\frac{p}{d^2}\right)}$$

$$= \frac{p}{Md^3}\sum_{\substack{m\neq m',m\neq n,\\ n\neq n',n'\neq m',\\ (m-m'-n+n')\bmod d=0}}\left|\sum_{\alpha=0}^{M-1}\omega^{\alpha(m^2-m'^2-n^2+n'^2)}\right|. \tag{S.27}$$

Using a variant of the Dirichlet kernel [15],

$$\sum_{\alpha=0}^{M-1} e^{i\alpha x} = e^{\frac{i(M-1)x}{2}}\frac{\sin\left(\frac{Mx}{2}\right)}{\sin\left(\frac{x}{2}\right)}, \quad \forall\ M\in\mathbb{N}, \tag{S.28}$$

and setting $x = \frac{2\pi}{d}(m^2-m'^2-n^2+n'^2)$, we have

$$\left|\sum_{\alpha=0}^{M-1}\omega^{\alpha(m^2-m'^2-n^2+n'^2)}\right| = \frac{\left|\sin\left(\frac{\pi M(m^2-m'^2-n^2+n'^2)}{d}\right)\right|}{\left|\sin\left(\frac{\pi(m^2-m'^2-n^2+n'^2)}{d}\right)\right|}. \tag{S.29}$$

Combining all terms of $\tilde{F}^{(M)} = F_1+\tilde{F}_2^{(M)}$, we obtain

$$\tilde{F}^{(M)} = 1-p+\frac{p}{d^2}-\frac{p}{Md^3}D_d^{(M)}, \tag{S.30}$$

where we define

$$D_d^{(M)} \coloneqq \sum_{\substack{m\neq m',m\neq n,\\ n\neq n',n'\neq m',\\ (m-m'-n+n')\bmod d=0}}\frac{\left|\sin\left(\frac{\pi M(m^2-m'^2-n^2+n'^2)}{d}\right)\right|}{\left|\sin\left(\frac{\pi(m^2-m'^2-n^2+n'^2)}{d}\right)\right|}. \tag{S.31}$$

For all odd prime d, $m^2-m'^2-n^2+n'^2\neq 0$ if m,m',n,n' satisfy all the constraints under the summation symbol in Eq. (S.31) [3]. Hence, $D_d^{(d)}=0$ for all odd prime d.

To witness the Schmidt number of $\rho^{\rm iso}$ to be at least $k+1$, $\tilde{F}^{(M)}$ must satisfy

$$\tilde{F}^{(M)} > \widetilde{\mathcal{B}}_k(|\psi\rangle = |\Phi_d^+\rangle) = \frac{k}{d}, \tag{S.32}$$

where $\widetilde{\mathcal{B}}_k(|\psi\rangle)$ is defined in Eq. (S.18). With some simple algebra, we see that Ineq. (S.32) is satisfied if and only if the white-noise ratio in $\rho^{\rm iso}$ satisfies

$$p < \frac{d(d-k)}{d^2-1+\frac{D_d^{(M)}}{Md}} \eqqcolon \tilde{p}^{(k)}_{{\rm iso},M} \quad \forall\ 1\leq M\leq d. \tag{S.33}$$

Since $D_d^{(M)}\geq 0$ for all $M,d\in\mathbb{N}$, $\tilde{p}^{(k)}_{{\rm iso},M}\leq p^{(k)}_{\rm iso}\coloneqq\frac{d(d-k)}{d^2-1}$ where $p^{(k)}_{\rm iso}$ is the minimum white-noise ratio for $\rho^{\rm iso}$ to have Schmidt number k [4]. Moreover, for odd-prime dimensions d, $\tilde{p}^{(k)}_{{\rm iso},M=d}=p^{(k)}_{\rm iso}$ since $D_d^{(d)}=0$.

S.II.3. Witnessing noisy purified thermal states

In this subsection, we will derive $\tilde{F}^{(M)} = F_1+\tilde{F}_2^{(M)}$ for the noisy thermal state $\rho_{\rm th}(p,\beta) = (1-p)|\psi_{\rm th}(\beta)\rangle\langle\psi_{\rm th}(\beta)| + \frac{p}{d^2}\mathbb{1}_{d^2}$ with $|\psi_{\rm th}(\beta)\rangle = \frac{1}{\sqrt{\mathcal{Z}}}\sum_{n=0}^{d-1}e^{-\frac{\beta n}{2}}|n\rangle\otimes|n\rangle$, where $\mathcal{Z}=\sum_{n=0}^{d-1}e^{-\beta n}$. Since $\rho_{\rm th}(p,\beta=0)=\rho^{\rm iso}$ and the corresponding fidelity bound $\tilde{F}^{(M)}$ and Schmidt-number witness bound $\widetilde{\mathcal{B}}_k(|\psi\rangle)$ as defined in Eq. (S.18) will be the same as in Sec. S.II.2, we will consider only the case with $\beta>0$ in this subsection. Knowing that

$$\langle ij|\rho_{\rm th}|ij\rangle = \frac{1-p}{\mathcal{Z}}e^{-\beta j}\delta_{ij}+\frac{p}{d^2} \quad \forall\ i,j\in[d], \tag{S.34}$$

the target state $|\psi\rangle$ has Schmidt coefficients

$$\lambda_j = \sqrt{\frac{(1-p)\frac{e^{-\beta j}}{\mathcal{Z}}+\frac{p}{d^2}}{1-p+\frac{p}{d}}} \tag{S.35}$$

according to Eq. (S.19). Note that the "tilted" bases in this case do not form an orthogonal bases for $\beta>0$. Using Eqs. (S.34) and (S.35), we can easily obtain

$$F_1 = \frac{1}{1-p+\frac{p}{d}}\left[\frac{(1-p)^2\tanh\frac{\beta}{2}}{\tanh\frac{d\beta}{2}}+\frac{2p(1-p)}{d^2}+\frac{p^2}{d^3}\right], \tag{S.36}$$

along with

$$\Sigma^{(M)} = \frac{d(1-p)}{\mathcal{Z}(\sum_j\lambda_j)^2}\left(\sum_{n=0}^{d-1}\lambda_n e^{-\frac{\beta n}{2}}\right)^2+\frac{p}{d}, \tag{S.37}$$

and

$$\sum_{m,n=0}^{d-1}\lambda_m\lambda_n\langle mn|\rho_{\rm th}|mn\rangle \tag{S.38}$$

$$= \frac{1-p}{1-p+\frac{p}{d}}\left[\frac{(1-p)\tanh\frac{\beta}{2}}{\tanh\frac{d\beta}{2}}+\frac{p}{d^2}\right]+\frac{p(\sum_j\lambda_j)^2}{d^2},$$

as well as the expressions

$$\sum_{\substack{m\neq m',m\neq n,\\ n\neq n',n'\neq m'}}\tilde{\gamma}^{(M)}_{mm'nn'}\sqrt{\langle m'n'|\rho_{\rm th}|m'n'\rangle\langle mn|\rho_{\rm th}|mn\rangle} = \frac{p}{Md^2}\overline{D}_d^{(M)}(p,\beta), \tag{S.39}$$

and finally

$$\overline{D}_d^{(M)}(p,\beta) \coloneqq \sum_{\substack{m\neq m',m\neq n,\\ n\neq n',n'\neq m',\\ (m-m'-n+n')\bmod d=0}}\sqrt{\lambda_m\lambda_n\lambda_{m'}\lambda_{n'}}\frac{\left|\sin\left(\frac{\pi M(m^2-m'^2-n^2+n'^2)}{d}\right)\right|}{\left|\sin\left(\frac{\pi(m^2-m'^2-n^2+n'^2)}{d}\right)\right|}, \tag{S.40}$$

where we again use the Dirichlet kernel [15] as in the previous subsection. Combining all terms of $\tilde{F}^{(M)} = F_1+$

$\tilde{F}_2^{(M)}$ and after some simplification, we obtain

$$\tilde{F}^{(M)} = \frac{p}{d^2}\left[1 - \frac{\overline{D}_d^{(M)}(p,\beta)}{M}\right] + (1-p)\kappa_d(p,\beta), \quad \text{(S.41)}$$

where $\kappa_d(p,\beta) := \frac{1}{\mathcal{Z}}\left(\sum_{n=0}^{d-1}\lambda_n e^{-\frac{\beta n}{2}}\right)^2$.

To witness the Schmidt number of $\rho_{\text{th}}(p,\beta)$ to be at least $k+1$, $\tilde{F}^{(M)}$ must satisfy $\tilde{F}^{(M)} > \widetilde{\mathcal{B}}_k(|\psi\rangle)$ where

$$\begin{aligned}\widetilde{\mathcal{B}}_k(|\psi\rangle) &= \sum_{j=0}^{k-1}\frac{(1-p)\frac{e^{-\beta j}}{\mathcal{Z}} + \frac{p}{d^2}}{1-p+\frac{p}{d}} \\ &= \frac{1}{1-p+\frac{p}{d}}\left[\frac{(1-p)(1-e^{-k\beta})}{1-e^{-d\beta}} + \frac{pk}{d^2}\right], \quad \text{(S.42)}\end{aligned}$$

as defined in Eq. (S.18). We can then numerically solve for the white-noise threshold ratio $\tilde{p}_{\text{th},M}^{(k)}$ such that $\tilde{F}^{(M)} > \widetilde{\mathcal{B}}_k(|\psi\rangle)$ is satisfied for all $p < \tilde{p}_{\text{th},M}^{(k)}$ (see Fig. S.7).

S.III. RANDOM MEASUREMENT BASES FOR WITNESSING SCHMIDT NUMBER IN HIGH DIMENSIONS

In this section, we will use concentration of measure to show that the likelihood of a set of orthonormal bases that are chosen uniformly at random in $\mathbb{C}^d$ to be biased decreases with the dimension d exponentially, i.e., proving Ineq. (12) in the main text.

First of all, we remark that there exists a bijection between all normalized pure states in $\mathbb{C}^d$ and the $(2d-1)$-sphere, $\mathbb{S}^{2d-1} = \{\vec{x} \in \mathbb{R}^{2d} : \|\vec{x}\|_2 = 1\}$ such that any normalized pure state in $\mathbb{C}^d$ can be written as

$$|\vec{x}\rangle = \sum_{k=0}^{d-1}(x_{2k+1} + ix_{2k+2})\,|k\rangle\,, \quad \text{(S.43)}$$

where $\vec{x} = \sum_{j=1}^{2d} x_j\vec{e}_j \in \mathbb{S}^{2d-1}$ and $\vec{e}_i^{\,T}\vec{e}_j = \delta_{ij}\ \forall\ i,j$.

Suppose that the first local measurement basis is $\{|i\rangle\}_{i=0}^{d-1}$ and the z-th basis with $z \in \{2,\dots,m\}$ is chosen to be $\{|e_a^z\rangle\}_{a=0}^{d-1}$ such that $|e_0^z\rangle \equiv |\vec{x}_0^{(z)}\rangle$ with $\vec{x}_0^{(z)}$ sampled uniformly at random on $\mathbb{S}^{2d-1}$ and $|e_a^z\rangle$ for $a = 1,\dots,d-1$ can be constructed by the Gram-Schmidt process using $\{|i\rangle\}_{i=0}^{d-1}$ as the reference basis. Let us define the set of rotation matrices $\{R_a^{(z)}\}_{a=1}^{d-1} \subset O(2d)$ such that $|e_a^z\rangle \equiv |\vec{x}_a^{(z)}\rangle \equiv |R_a^{(z)}\vec{x}_0^{(z)}\rangle$. Using the bijection in Eq. (S.43), the overlap between the first and the z-th bases is given by

$$f_j(\vec{x}_a^{(z)}) := |\langle j|\vec{x}_a^{(z)}\rangle|^2 = (x_a^{(z)})_{2j+1}^2 + (x_a^{(z)})_{2j+2}^2 \quad \text{(S.44)}$$

for $a, j \in \{0,\dots,d-1\}$.

To find the average of the bases overlap $\mathbb{E}_{\mathbb{S}^{2d-1}}[f_j(\vec{x}_a^{(z)})]$, we integrate $f_j(\vec{x}_a^{(z)})$ over $\mathbb{S}^{2d-1}$ in the spherical coordinates with respect to the uniform spherical measure μ on $\mathbb{S}^{2d-1}$. Since μ is invariant under any rotation $R \in O(2d)$ and $\{\vec{x}_a^{(z)}\}_{a,z}$ are all related by some rotations in $O(2d)$,

$$\mathbb{E}_{\mathbb{S}^{2d-1}}[f_j(\vec{x}_a^{(z)})] = \mathbb{E}_{\mathbb{S}^{2d-1}}[f_j(\vec{x})] \quad \forall\ a,z, \quad \text{(S.45)}$$

where $\vec{x} \in \mathbb{S}^{2d-1}$. Hence, we can drop the indices a and z in the integral. As a remark, the average $\mathbb{E}_{\mathbb{S}^{2d-1}}[f_j(\vec{x})]$ is not affected if $|\vec{x}\rangle$ is defined in another basis, i.e., $|\vec{x}\rangle = \sum_{k=0}^{d-1}(x_{2k+1} + ix_{2k+2})\,|\tilde{k}\rangle$ where $\{|i\rangle\}_i \neq \{|\tilde{k}\rangle\}_k$, as there exist $\vec{x}' = R\vec{x} \in \mathbb{S}^{2d-1}$ with $R \in O(2d)$ such that $|\vec{x}\rangle = \sum_{k=0}^{d-1}(x'_{2k+1} + ix'_{2k+2})\,|k\rangle$.

Moreover, the average overlap between the z-th and z'-th bases for any $z, z' \in \{2,\dots,m\}$ also equals to $\mathbb{E}_{\mathbb{S}^{2d-1}}[f_j(\vec{x})]$. To illustrate that, we consider the overlap $|\langle\vec{x}_a^{(z)}|\vec{x}_b^{(z')}\rangle|^2$. Since there exists a unitary $U \in U(d)$ such that $U|\vec{x}_a^{(z)}\rangle = |j\rangle$ and $U|\vec{x}_b^{(z')}\rangle = |R_U\vec{x}_b^{(z')}\rangle$ where $R_U \in O(2d)$ and $R_U\vec{x}_b^{(z')} \in \mathbb{S}^{2d-1}$, we have

$$|\langle\vec{x}_a^{(z)}|\vec{x}_b^{(z')}\rangle|^2 = |\langle\vec{x}_a^{(z)}|U^\dagger U|\vec{x}_b^{(z')}\rangle|^2 = |\langle j|R_U\vec{x}_b^{(z')}\rangle|^2, \quad \text{(S.46)}$$

and the average of the bases overlap is

$$\begin{aligned}&\int_{\vec{x}_b^{(z')}\in\mathbb{S}^{2d-1}} |\langle\vec{x}_a^{(z)}|\vec{x}_b^{(z')}\rangle|^2 d\mu(\mathbb{S}^{2d-1}) \quad \text{(S.47)}\\ &= \int_{\vec{x}_b^{(z')}\in\mathbb{S}^{2d-1}} |\langle j|R_U\vec{x}_b^{(z')}\rangle|^2 d\mu(\mathbb{S}^{2d-1}) \\ &= \int_{\vec{x}_b^{(z')}\in\mathbb{S}^{2d-1}} |\langle j|\vec{x}_b^{(z')}\rangle|^2 d\mu(R_U^{-1}\mathbb{S}^{2d-1}) \\ &= \int_{\vec{x}_b^{(z')}\in\mathbb{S}^{2d-1}} f_j(\vec{x}_b^{(z')}) d\mu(\mathbb{S}^{2d-1}) \\ &= \mathbb{E}_{\mathbb{S}^{2d-1}}[f_j(\vec{x}_b^{(z')})] = \mathbb{E}_{\mathbb{S}^{2d-1}}[f_j(\vec{x})]\,,\end{aligned}$$

where we use the invariance of the spherical measure $\mu(R_U^{-1}\mathbb{S}^{2d-1}) = \mu(\mathbb{S}^{2d-1})$ and Eq. (S.45). Therefore, the overlaps between all m bases (with $m-1$ randomly chosen ones) will all follow the same statistics, so we can consider only the overlaps between the first two bases from now on without loss of generality.

To perform the integration for averaging $f_j(\vec{x})$, we use the standard conversion between the Cartesian, of which $\vec{x}$ is written in Eqs. (S.43) and (S.44), and the spherical coordinates [16] on a unit sphere, i.e.,

$$x_{1\le j\le 2d-1} = \cos(\varphi_j)\prod_{i=1}^{j-1}\sin(\varphi_i), \quad \text{(S.48a)}$$

$$x_{2d} = \prod_{i=1}^{2d-1}\sin(\varphi_i), \quad \text{(S.48b)}$$

where $\varphi_1,\dots,\varphi_{2d-2} \in [0,\pi]$ and $\varphi_{2d-1} \in [0,2\pi)$. Using the expression of the surface area element of $\mathbb{S}^{2d-1}$ in the spherical coordinates [16],

$$dA_{\mathbb{S}^{2d-1}} = d\varphi_{2d-1}\prod_{i=1}^{2d-2}\sin^{2d-i-1}\varphi_i d\varphi_i, \quad \text{(S.49)}$$

together with the identities

$$\int_0^\pi \sin^\alpha\theta\, d\theta = \sqrt{\pi}\frac{\Gamma\left(\frac{1+\alpha}{2}\right)}{\Gamma\left(1+\frac{\alpha}{2}\right)} \quad \forall\ \mathrm{Re}(\alpha) > -1, \quad \text{(S.50)}$$

and

$$\prod_{j=1}^{k}\frac{\Gamma\left(d-\frac{j}{2}\right)}{\Gamma\left(d+1-\frac{j+1}{2}\right)} = \frac{\Gamma\left(d-\frac{k}{2}\right)}{\Gamma(d)}, \quad \text{(S.51)}$$

with $\Gamma(n) = (n-1)!\ \forall\ n \in \mathbb{N}$, the total area of $\mathbb{S}^{2d-1}$ is

$$A_{\mathbb{S}^{2d-1}} = \prod_{i=1}^{2d-2} \int_0^{\pi} \sin^{2d-i-1}\varphi_i d\varphi_i \int_0^{2\pi} d\varphi_{2d-1} = \frac{2\pi^d}{(d-1)!}\,. \tag{S.52}$$

Next, we use Eqs. (S.48a) and (S.48b) to write

$$f_j(\vec{x}) = \prod_{i=1}^{2j} \sin^2\varphi_i (1 - \sin^2\varphi_{2j+1}\sin^2\varphi_{2j+2}) \tag{S.53}$$

in the spherical coordinates, where we define $\varphi_{2d} = 0$, and by using

$$\prod_{j=1}^{2k} \frac{\Gamma\left(d+1-\frac{j}{2}\right)}{\Gamma\left(d+1-\frac{j-1}{2}\right)} = \frac{(d-k)!}{d!}, \tag{S.54}$$

we can calculate the average bases overlap to be

$$\mathbb{E}_{\mathbb{S}^{2d-1}}[f_j(\vec{x})] = \int_{\mathbb{S}^{2d-1}} f_j(\vec{x}) \frac{dA_{\mathbb{S}^{2d-1}}}{A_{\mathbb{S}^{2d-1}}} = \frac{1}{d} \tag{S.55}$$

for all $j \in \{0, \ldots, d-1\}$. This shows that the average bases overlaps over the uniform spherical measure are all equal to the bases overlaps of MUBs.

Next, we apply Lévy's lemma which upper bounds the likelihood that the overlaps between a randomly chosen basis and the fixed basis or between two randomly chosen bases deviate from $\frac{1}{d}$ by $\epsilon > 0$.

Lemma S.1 (Lévy's Lemma [17–19])**.** *Let $f : \mathbb{S}^{d'-1} \mapsto \mathbb{R}$ be a Lipschitz continuous function such that $\exists\ K \geq 0$,*

$$|f(\vec{x}) - f(\vec{y})| \leq K\|\vec{x} - \vec{y}\|_2 \quad \forall\ \vec{x}, \vec{y} \in \mathbb{S}^{d'-1}. \tag{S.56}$$

For a point $\vec{x} \in \mathbb{S}^{d'-1}$ chosen uniformly at random,

$$Pr\{|f(\vec{x}) - \mathbb{E}[f]| > \epsilon\} \leq 2\exp\left(-\frac{Cd'\epsilon^2}{K^2}\right), \tag{S.57}$$

where $\mathbb{E}[f]$ is the mean value of f and the constant $C > 0$ may take the value $C = (9\pi^3 \ln 2)^{-1}$.

In order to use Lévy's lemma, we must show that $f_j(\vec{x})$ is Lipschitz continuous. To show this, we return to the Cartesian coordinate and define $P_{i,j} = \vec{e}_i \cdot \vec{e}_i^{\,T} + \vec{e}_j \cdot \vec{e}_j^{\,T}$ for $i \neq j$, $X = \vec{x} \cdot \vec{x}^T$, and $Y = \vec{y} \cdot \vec{y}^T$. For any $\vec{x}, \vec{y} \in \mathbb{S}^{2d-1}$,

$$\begin{aligned} |f_j(\vec{x}) - f_j(\vec{y})| &= |x_{2j+1}^2 + x_{2j+2}^2 - y_{2j+1}^2 - y_{2j+2}^2| \\ &= |\mathrm{Tr}[(X - Y)P_{2j+1,2j+2}]| \\ &\leq \|X - Y\|_{\mathrm{HS}}\|P_{2j+1,2j+2}\|_{\mathrm{HS}}, \end{aligned} \tag{S.58}$$

where we use the Cauchy–Schwarz inequality and the Hilbert-Schmidt norm, $\|\cdot\|_{\mathrm{HS}}$. Since $\|P_{i,j}\|_{\mathrm{HS}} = \sqrt{2}$,

$$\begin{aligned} |f_j(\vec{x}) - f_j(\vec{y})| &\leq \sqrt{2\,\mathrm{Tr}[(X-Y)^\dagger(X-Y)]} \\ &= \sqrt{2[(\vec{x}^T\vec{x})^2 + (\vec{y}^T\vec{y})^2 - 2|\vec{x}^T\vec{y}|^2]} \\ &= 2\sqrt{1 - |\vec{x}^T\vec{y}|^2}, \end{aligned} \tag{S.59}$$

where we use the fact that $\vec{x}, \vec{y} \in \mathbb{S}^{2d-1}$. Then, knowing that $\vec{x}^T\vec{y} = \sum_{j=1}^{2d} x_j y_j \leq 1$, we have

$$\begin{aligned} |f_j(\vec{x}) - f_j(\vec{y})| &\leq 2\sqrt{\left(1 - \sum_{i=1}^{2d} x_i y_i\right)\left(1 + \sum_{j=1}^{2d} x_j y_j\right)} \\ &\leq 2\sqrt{2\left(1 - \sum_i x_i y_i\right)} = 2\|\vec{x} - \vec{y}\|_2\,. \end{aligned} \tag{S.60}$$

Hence, the Lipschitz constant in Eq. (S.56) is $K = 2$ and by setting $d' = 2d$, Lévy's lemma tells us that

$$\Pr\left\{\left|f_j(\vec{x}) - \frac{1}{d}\right| > \epsilon\right\} \leq 2\exp\left(-\frac{d\epsilon^2}{18\pi^3 \ln 2}\right). \tag{S.61}$$

This implies that in large dimensions d, it is very likely to find two randomly chosen bases to be close to mutually unbiased. Since we can still certify entanglement in high dimensions with measurement bases that are not MUBs with our witness, Ineq. (S.61) suggests that it may not worth the effort to precisely control the relative phases of the measurement bases to ensure that they are mutually unbiased in large dimensions.

S.IV. MAXIMAL NUMBER OF ORTHONORMAL BASES WITH OVERLAP CONSTRAINTS

In this section, we will prove the following corollary which relates the maximal number of orthonormal bases that obey certain overlap constraints to the function $\lambda(\mathcal{C})$ defined in Theorem 1.

Corollary S.1. *Suppose that there are m orthonormal bases with overlaps $\mathcal{C} = \{|\langle e_a^z | e_{a'}^{z'}\rangle|^2\}_{a,a',z\neq z'}$. Then,*

$$m \leq \frac{d+1}{2}\left(1 + \sqrt{1 + \frac{8\lambda(\mathcal{C})\big(\lambda(\mathcal{C}) - 1\big)}{d^2 - 1}}\right) =: \overline{m}_d \tag{S.62}$$

for all $d \geq 2$, where $\lambda(\mathcal{C})$ is defined in Theorem 1.

We first give the formal statement of the Welch bounds before proving the corollary. These bounds limit the maximal number of unit vectors compatible with certain overlaps between them.

Lemma S.2 (Welch bounds [20])**.** *Let $\{|\psi_i\rangle\}_{i=1}^M \subset \mathbb{C}^d$ be a set of M unit vectors. Then, for all $k \geq 1$,*

$$\sum_{i,j=1}^{M} |\langle\psi_i|\psi_j\rangle|^{2k} \geq \frac{M^2}{\binom{d+k-1}{k}}. \tag{S.63}$$

Proof of Corollary S.1. Our goal is to upper bound the number of orthonormal bases, m, for a given value of $\lambda(\mathcal{C})$ that is defined in Theorem 1. Let us apply Lemma S.2

by setting $M = md$ and $\{|\psi_i\rangle\}_{i=1}^{M} = \{|e_a^z\rangle\}_{a,z}$. The summation term in Ineq. (S.63) can be split into two terms

$$\sum_{i,j=1}^{M} |\langle\psi_i|\psi_j\rangle|^{2k} = \sum_z \sum_{a,a'} |\langle e_a^z|e_{a'}^z\rangle|^{2k} + \sum_{z\neq z'} \sum_{a,a'} |\langle e_a^z|e_{a'}^{z'}\rangle|^{2k}. \tag{S.64}$$

Now we set $k = 2$, then the first term evaluates to $\sum_z \sum_{a,a'} |\delta_{a,a'}|^4 = md$ and the second term is related to $\lambda(\mathcal{C})$ by

$$\sum_{\substack{z\neq z'\\ a,a'}} |\langle e_a^z|e_{a'}^{z'}\rangle|^4 = 2\lambda(\mathcal{C})\big(\lambda(\mathcal{C})-1\big) + d\sum_{z\neq z'}\left[(d+1)c_{\min}^{z,z'} - 1\right]. \tag{S.65}$$

Using the fact that $c_{\min}^{z,z'} \leq \frac{1}{d}\ \forall\ z, z'$ and after some algebra, we obtain

$$m^2 - (d+1)m - \frac{2(d+1)}{d-1}\lambda(\mathcal{C})\big(\lambda(\mathcal{C})-1\big) \leq 0, \tag{S.66}$$

which implies the inequality

$$m \leq \frac{d+1}{2}\left(1 + \sqrt{1 + \frac{8\lambda(\mathcal{C})\big(\lambda(\mathcal{C})-1\big)}{d^2-1}}\right) =: \overline{m}_d \tag{S.67}$$

for all $d \geq 2$, as stated in Corollary S.1. □

S.V. PROOF OF LEMMA 2

In this section, we present the proof of Lemma 2 from the main article, which we restate here for clarity.

Lemma 2. *For any $d \in \mathbb{N}$, the three orthonormal bases $\{|e_a^1\rangle = |a\rangle\}_{a=0}^{d-1}$, $\{|e_a^2\rangle\}_{a=0}^{d-1}$, and $\{|e_a^3\rangle\}_{a=0}^{d-1}$, with*

$$|e_a^2\rangle = \frac{1}{\sqrt{d}}\sum_{j=0}^{d-1} e^{i2\pi\left[\frac{aj}{d}+f(j)\right]}|j\rangle, \tag{S.68a}$$

$$|e_a^3\rangle = \frac{1}{\sqrt{d}}\sum_{j=0}^{d-1} e^{i2\pi\left[\frac{(d-p^r)j^2}{2d}+\frac{aj}{d}+f(j)\right]}|j\rangle, \tag{S.68b}$$

where f is any real-valued function, $r \in \mathbb{N}\cup\{0\}$, and p is any odd prime such that $gcd(d,p) = 1$ and $d > p^r$, are mutually unbiased. The simplest example would be having $p^r = 1$.

We remark that the freedom in defining the relative phases $\{e^{i2\pi f(j)}\}_j$ in both bases can be thought of as the global phase freedom in defining the computational basis. We also note that the quadratic phases in our construction and our bases unbiasedness can be related to properties of stabilizer states in odd dimensions [21, 22].

To prove this, we will need the following propositions and observations, where Propositions S.1 and S.2 were proven in Refs. [23] and [24], respectively.

Proposition S.1 (Lemma 1 in Ref. [23]). *Let $a, b \in \mathbb{Z}$ and c be a positive odd integer such that $gcd(a,c) = 1$. Then,*

$$G(a,b,c) := \sum_{n=0}^{c-1} e^{2\pi i\frac{an^2+bn}{c}} = \varepsilon_c\sqrt{c}\left(\frac{a}{c}\right)e^{-2\pi i\frac{\psi(a)b^2}{c}}, \tag{S.69}$$

where $\left(\frac{a}{c}\right) \in \{-1,0,1\}$ is the Jacobi symbol,

$$\varepsilon_c = \begin{cases}1 & \text{if } c\equiv 1 \mod 4,\\ i & \text{if } c\equiv 3 \mod 4,\end{cases} \tag{S.70}$$

and $\psi(a) \in \mathbb{Z}$ such that $4\psi(a)a \equiv 1 \mod c$. Therefore, $|G(a,b,c)| = \sqrt{c}$.

Proposition S.2 (Reciprocity theorem for generalized Gauss sums [Theorem 1.2.2 in Ref. [24]]). *Let $a, b, c \in \mathbb{Z}$ such that $ac \neq 0$ and $ac+b$ is even. Then,*

$$S(a,b,c) := \sum_{n=0}^{|c|-1} e^{i\pi\frac{an^2+bn}{c}} = \left|\frac{c}{a}\right|^{\frac{1}{2}} e^{i\pi\frac{|ac|-b^2}{4ac}} S(-c,-b,a). \tag{S.71}$$

Observation S.1. *For all odd $d \in \mathbb{N}$, odd prime p such that $gcd(d,p) = 1$ and $r \in \mathbb{N}\cup\{0\}$, $gcd\left(d, \frac{d-p^r}{2}\right) = 1$.*

Proof. For odd $d \in \mathbb{N}$, $\frac{d-p^r}{2} \in \mathbb{Z}$. Let $gcd\left(d,\frac{d-p^r}{2}\right) = k$. Then, there exist $n, m \in \mathbb{N}$ such that $d = nk$ and $\frac{d-p^r}{2} = mk$. Since $k \neq p^q$ for any $q \in \mathbb{N}$ due to $gcd(d,p) = 1$, it follows that the largest $k \in \mathbb{N}$ that satisfies $(n-2m)k = d-(d-p^r) = p^r$ is $k = 1$. □

Observation S.2. *For all even $d \in \mathbb{N}$, odd prime p such that $gcd(d,p) = 1$ and $r \in \mathbb{N}\cup\{0\}$, $gcd\left(d-p^r, \frac{d}{2}\right) = 1$.*

Proof. Let $gcd\left(d-p^r, \frac{d}{2}\right) = k$. Then, there exist $n, m \in \mathbb{N}$ such that $d-p^r = nk$ and $\frac{d}{2} = mk$. Since $k \neq p^q$ for any $q \in \mathbb{N}$ due to $gcd(d,p) = 1$, the largest $k \in \mathbb{N}$ that satisfies $(2m-n)k = d-(d-p^r) = p^r$ is $k = 1$. □

Proposition S.3. *Let $d \in \mathbb{N}$, $k \in \mathbb{Z}$, $r \in \mathbb{N}\cup\{0\}$, and p be an odd prime such that $gcd(d,p) = 1$ and $d > p^r$. Then, it holds that*

$$\mathcal{G}_d^k := \left|\sum_{j=0}^{d-1} e^{2\pi i\left(\frac{(d-p^r)j^2}{2d}+\frac{kj}{d}\right)}\right| = \sqrt{d}. \tag{S.72}$$

Proof. For all odd $d \in \mathbb{N}$, $d-p^r$ is even and $gcd\left(d, \frac{d-p^r}{2}\right) = 1$ by Observation S.1, so we can apply Proposition S.1 to get

$$\mathcal{G}_d^k = \left|\sum_{j=0}^{d-1} e^{i2\pi\frac{\left(\frac{d-p^r}{2}\right)j^2+kj}{d}}\right| = \left|G\left(\frac{d-p^r}{2}, k, d\right)\right| = \sqrt{d}. \tag{S.73}$$

We now consider the remaining cases with even $d \in \mathbb{N}$. The reciprocity theorem (Proposition S.2) can be applied in our case for all even $d \in \mathbb{N}_{\geq 2}$ because if we let

$a = d - p^r$, $b = 2k$, and $c = d$, then $ac \neq 0$ $\forall$ even $d > 1$ and $ac + b = d(d - p^r) + 2k$ is even for all $d, k \in \mathbb{Z}$, odd p, and $r \in \mathbb{N} \cup \{0\}$.

For all even $d \in \mathbb{N}$, we apply Proposition S.2 to get

$$\mathcal{G}_d^k = |S(d - p^r, 2k, d)| = \sqrt{\frac{d}{d - p^r}} |S(-d, -2k, d - p^r)|, \tag{S.74}$$

where we observe that

$$S(-d, -2k, d - p^r) = \sum_{j=0}^{d-p^r-1} e^{-2\pi i \frac{(\frac{d}{2})j^2 + kj}{d-p^r}} = G\left(-\frac{d}{2}, -k, d - p^r\right). \tag{S.75}$$

Since $d - p^r$ is odd and $gcd\left(d - p^r, \frac{d}{2}\right) = 1$ by Observation S.2, we can apply Proposition S.1 to get $\left|G\left(-\frac{d}{2}, -k, d - p^r\right)\right| = \sqrt{d - p^r}$. Combining this with Eqs. (S.74) and (S.75), we obtain $\mathcal{G}_d^k = \sqrt{d}$ for all even $d \geq 2$ as well. □

Finally, we can state the proof of Lemma 2 having proven Proposition S.3.

Proof of Lemma 2. First of all, all three bases are clearly orthonormal as we know that $\frac{1}{d} \sum_{j=0}^{d-1} e^{i2\pi \frac{(b-a)j}{d}} = \delta_{a,b}$ for all $a, b \in \mathbb{Z}$ and $d \in \mathbb{N}_{\geq 2}$. It is obvious that $|\langle e_a^1 | e_b^z \rangle| = \frac{1}{\sqrt{d}}$ for all $z \in \{2, 3\}$ and $a, b \in [d] := \{0, \ldots, d-1\}$. To prove that $|\langle e_a^2 | e_b^3 \rangle| = \frac{1}{\sqrt{d}}$ for all $a, b \in [d]$, it follows that

$$|\langle e_a^2 | e_b^3 \rangle| = \frac{1}{d} \left| \sum_{j=0}^{d-1} e^{i2\pi \left(\frac{(d-p^r)j^2}{2d} + \frac{(b-a)j}{d} \right)} \right| = \frac{1}{\sqrt{d}}, \tag{S.76}$$

where the last equality follows from Proposition S.3 if we set the integer $k = b - a$. □

S.VI. APPLICATION OF AMUBS

In this section, we investigate whether using approximately MUBs (AMUBs) as the local measurement bases can provide any practical advantage in witnessing Schmidt number in local dimensions where the maximum number of MUBs is unknown.

Suppose that we can control an arbitrary number of measurement bases with extreme precision. As Corollary 1 suggests, we should aim at locally measuring in as many MUBs as possible to get the best performance of our witness. Sadly, the total number of MUBs is unknown for dimensions that are not prime powers (e.g., $d = 6, 10, 12$). In fact, given the prime factorization of the dimension $d = \prod_j p_j^{n_j}$ with $p_j^{n_j} < p_{j+1}^{n_{j+1}}$ $\forall$ j, (tensor products of) the Wootters–Fields construction only guarantees $p_1^{n_1} + 1$ MUBs to exist [25]. However, even with such construction, calculating all the relative phases for each basis is non-trivial for large prime powers [14].

Alternatively, if we allow our measurement bases to be nearly mutually unbiased, then one can construct $d + 1$ AMUBs for any dimension d [26]. The AMUBs construction in Ref. [26] has a simpler description than the one by Wootters–Fields and takes the following form,

$$|M_a^z\rangle = \frac{1}{\sqrt{d}} \sum_{j=1}^{d} \exp\left[i2\pi \left(\frac{zj^2}{p} + \frac{aj}{d} \right) \right] |j - 1\rangle, \tag{S.77}$$

where $z \in \{1, \ldots, d\}$ labels the basis, $a \in [d]$ labels the basis vector, and p is the smallest prime such that $p \geq d$. Together with the standard basis $\{|M_j^0\rangle = |j\rangle\}_j$, they form a set of $d+1$ AMUBs. Using a result from Ref. [27], it was shown that the bases overlaps satisfy [26]

$$|\langle M_a^z | M_{a'}^{z'} \rangle|^2 < \frac{4p}{\pi d^2} \left(\ln p + \gamma - \ln \frac{\pi}{2} + \frac{\pi - 1}{2p} \right) + \frac{1}{d} \tag{S.78}$$

for all $z \neq z' \in \{1, \ldots, d\}$ and $a, a' \in [d]$, where $\gamma \approx 0.577$ is Euler's constant. Also, we have $|\langle M_a^z | j \rangle|^2 = \frac{1}{d}$ for all $z \in \{1, \ldots, d\}$ and $a, j \in [d]$. By the Bertrand–Chebyshev theorem [28, 29], which says that there exists a prime p such that $n < p \leq 2n$ for all $n \in \mathbb{N}$, we get

$$c_{\max}^{z,z'} < \frac{8}{\pi d} \left(\ln(2d) + \gamma - \ln \frac{\pi}{2} + \frac{\pi - 1}{4d} \right) + \frac{1}{d} \tag{S.79}$$

for $z \neq z' \in \{1, \ldots, d\}$, which scales as $\frac{8 \ln d}{\pi d} + \mathcal{O}\left(\frac{1}{d}\right)$. Note that the upper bound in Ineq. (S.79) is larger than 1 for $d < 9$, so the bound is only relevant for large dimensions. In large local dimensions d, since we are not aware of any non-trivial analytic lower bound for $c_{\min}^{z,z'}$, we have to assume $c_{\min}^{z,z'} = 0$ and due to the loose upper bound for $c_{\max}^{z,z'}$ in Ineq. (S.79), we cannot witness any non-trivial Schmidt number of any state using Lemma 1 as $\overline{\mathcal{T}}(\overline{\mathcal{C}}) = m$. For small local dimensions d, one can easily find the smallest prime $p \geq d$ [30], so the values of $c_{\max}^{z,z'}$ and $c_{\min}^{z,z'}$ should be computable.

To see whether using AMUBs as the local measurement bases in our Schmidt-number witness construction can provide any advantage when the maximal set of MUBs is not known, we consider the smallest non-prime-powered dimensions with the least known MUBs: $d = 6, 10, 14,$ and 22 [31]. The smallest primes $p \geq d$ that enter into Eq. (S.77) are 7, 11, 17, and 23, respectively. Our goal is to see if using $4 \leq m \leq d + 1$ of these AMUBs can witness higher Schmidt numbers than using only the 3 known MUBs. For this purpose, we consider the isotropic state (see Sec. S.I.1) again and compare the noise tolerances for witnessing each Schmidt number $2 \leq k \leq d$ using $4 \leq m \leq d + 1$ AMUBs with those using only 3 MUBs. Unfortunately, we verify numerically that measuring in any $m \in \{4, \ldots, d + 1\}$ AMUBs described by Eq. (S.77) gives zero noise tolerance. Interestingly, we find that by replacing p in Eq. (S.77) with a non-prime value can sometimes improve the noise tolerance slightly. For example, in $d = 6$, if $p = 7$ is replaced by 7.2, then measuring in a particular subset of 4 "modified" AMUBs will give

strictly positive noise tolerances for all $k \in \{2, \dots, 6\}$, but still not enough to surpass the noise tolerance when using 3 MUBs. In fact, by replacing $p = 7$ with 7.2, three of the bases become mutually unbiased. In Sec. S.V, we generalize this observation, enabling us to construct 3 MUBs with a simple analytic description in any dimension $d \in \mathbb{N}$. This observation also suggests that there could be other constructions of AMUBs that are more suited for witnessing Schmidt numbers than the one from Ref. [26].

---

[1] Charles H. Bennett, David P. Di Vincenzo, John A. Smolin, and William K. Wootters, *Mixed-state entanglement and quantum error correction,* Phys. Rev. A **54**, 3824 (1996), arXiv:quant-ph/9604024.

[2] Michał Horodecki, Paweł Horodecki, and Ryszard Horodecki, *General teleportation channel, singlet fraction and quasidistillation,* Phys. Rev. A **60**, 1888 (1999), arXiv:quant-ph/9807091.

[3] Jessica Bavaresco, Natalia Herrera Valencia, Claude Klöckl, Matej Pivoluska, Paul Erker, Nicolai Friis, Mehul Malik, and Marcus Huber, *Measurements in two bases are sufficient for certifying high-dimensional entanglement,* Nat. Phys. **14**, 1032–1037 (2018), arXiv:1709.07344.

[4] Barbara M. Terhal and Paweł Horodecki, *Schmidt number for density matrices,* Phys. Rev. A **61**, 040301(R) (2000), arXiv:quant-ph/9911117.

[5] As it is harder to witness a state to have Schmidt number $k+1$ with a larger bound $\mathcal{B}_k$, this is the worst-case bases choice for a given $c_{\min} = \min_{z,z'} c_{\min}^{z,z'}$ because (i) it gives the maximal value allowed for $c_{\max} = \max_{z,z'} c_{\max}^{z,z'}$ such that $\sum_{a'} |\langle e_a^z | e_{a'}^{z'} \rangle|^2 = 1$ holds for all a, and (ii) $\mathcal{T}(\mathcal{C})$ (and therefore $\mathcal{B}_k$) increases with $c_{\max}$ for a fixed $c_{\min}$ due to the enlargement of the feasible set of the optimization problem in Proposition 3 (see Methods in main article).

[6] The Schmidt-number witness in Ref. [3] is proven to be optimal only for all pure states and dephased maximally entangled states, $(1-p)\,|\Phi_d^+\rangle\langle\Phi_d^+| + \frac{p}{d}\sum_{i=0}^{d-1} |ii\rangle\langle ii|$.

[7] Note that we plot $\sqrt{\Delta\mathcal{F}_m^{\text{iso}}}$ instead of $\Delta\mathcal{F}_m^{\text{iso}}$ for clearer presentation because the curves appear too close to one another when we plot $\Delta\mathcal{F}_m^{\text{iso}}$ versus p.

[8] I. D. Ivonović, *Geometrical description of quantal state determination,* J. Phys. A: Math. Gen. **14**, 3241 (1981).

[9] The only θ-dependent term in Eq. (S.15) is the quantity $\overline{\mathcal{T}}(\overline{\mathcal{C}})$ which depends only on $c_{\min}^{z,z'}$ and $c_{\max}^{z,z'}$ (see Lemma 1). Since $c_{\min}^{z,z'}$ and $c_{\max}^{z,z'}$ in Eqs. (S.11a) and (S.13) are invariant under $\theta \leftrightarrow 2\pi - \theta$ for all $z \neq z'$, $p_{\text{th},m}^{(k)}(\beta,\theta) = p_{\text{th},m}^{(k)}(\beta, 2\pi-\theta)$.

[10] Since any unitary U has a spectral decomposition $U = \sum_i \lambda_i |\lambda_i\rangle\langle\lambda_i|$, where all eigenvalues satisfy $|\lambda_i| = 1$ and $\{|\lambda_i\rangle\}_i$ is an orthonormal basis, any matrix element of U satisfies $|U_{jk}| = |\sum_i \lambda_i \langle j|\lambda_i\rangle\langle\lambda_i|k\rangle| \leq \sqrt{\sum_i |\lambda_i| |\langle j|\lambda_i\rangle|^2}\sqrt{\sum_i |\lambda_i| |\langle k|\lambda_i\rangle|^2} = 1$, where we use the Cauchy–Schwarz inequality.

[11] Kai-Chi Chang, Murat C. Sarihan, Xiang Cheng, Paul Erker, Nicky Kai Hong Li, Andrew Mueller, Maria Spiropulu, Matthew D. Shaw, Boris Korzh, Marcus Huber, and Chee Wei Wong, *Experimental high-dimensional entanglement certification and quantum steering with time-energy measurements,* arXiv:2310.20694 (2024).

[12] Niklas Euler and Martin Gärttner, *Detecting high-dimensional entanglement in cold-atom quantum simulators,* PRX Quantum **4**, 040338 (2023), arXiv:2305.07413.

[13] Note that for all odd-prime dimensions d, $\tilde{F}^{(M')} \geq \tilde{F}^{(M)}$ holds for all $M' \geq M$, whereas for all other dimensions, only $\tilde{F}^{(M)} \geq \tilde{F}^{(1)}$ is guaranteed to hold $\forall\ M \geq 1$ [3].

[14] William K. Wootters and Brian D. Fields, *Optimal state-determination by mutually unbiased measurements,* Ann. Phys. **191**, 363–381 (1989).

[15] Johann Peter Gustav Lejeune Dirichlet, *Sur la convergence des séries trigonométriques qui servent à représenter une fonction arbitraire entre des limites données.* Journal für die reine und angewandte Mathematik **4**, 157–169 (1829), http://eudml.org/doc/183134.

[16] L. E. Blumenson, *A Derivation of n-Dimensional Spherical Coordinates,* Am. Math. Mon. **67**, 63–66 (1960).

[17] Vitali D. Milman and Gideon Schechtman, *Asymptotic Theory of Finite Dimensional Normed Spaces*, Lecture Notes in Mathematics (Springer, Berlin, Heidelberg, 1986).

[18] Michel Ledoux, *The Concentration of Measure Phenomenon*, Mathematical Surveys and Monographs, Vol. 89 (American Mathematical Society, Providence, Rhode Island, 2001).

[19] Patrick Hayden, Debbie W. Leung, and Andreas Winter, *Aspects of generic entanglement,* Commun. Math. Phys. **265**, 95–117 (2006), arXiv:0407049.

[20] Lloyd R. Welch, *Lower bounds on the maximum cross correlation of signals (Corresp.),* IEEE Trans. Inf. Theory **20**, 397–399 (1974).

[21] David Gross, *Hudson's theorem for finite-dimensional quantum systems,* J. Math. Phys. **47**, 122107 (2006), arXiv:quant-ph/0602001.

[22] Richard Kueng and David Gross, *Qubit stabilizer states are complex projective 3-designs,* arXiv:1510.02767 [quant-ph] (2015).

[23] Natalia Herrera Valencia, Vatshal Srivastav, Matej Pivoluska, Marcus Huber, Nicolai Friis, Will McCutcheon, and Mehul Malik, *High-Dimensional Pixel Entanglement: Efficient Generation and Certification,* Quantum **4**, 376 (2020), arXiv:2004.04994.

[24] Bruce C. Berndt, Ronald J. Evans, and Kenneth S. Williams, *Gauss and Jacobi Sums*, Wiley-Interscience and Canadian Mathematics Series of Monographs and Texts (Wiley, New York, 1998).

[25] Maria Prat Colomer, Luke Mortimer, Irénée Frérot, Máté Farkas, and Antonio Acín, *Three numerical approaches to find mutually unbiased bases using Bell inequalities,* Quantum **6**, 778 (2022), arXiv:2203.09429 [quant-ph].

[26] Igor E. Shparlinski and Arne Winterhof, *Constructions of approximately mutually unbiased bases,* in *LATIN 2006: Theoretical Informatics*, edited by José R. Correa, Alejandro Hevia, and Marcos Kiwi (Springer, Berlin, Heidelberg, 2006) p. 793–799.

[27] Todd Cochrane and J. C. Peral, *An Asymptotic Formula*

for a Trigonometric Sum of Vinogradov, J. Number Theory **91**, 1–19 (2001).

[28] Joseph Bertrand, *Mémoire sur le nombre de valeurs que peut prendre une fonction quand on y permute les lettres qu'elle renferme.* Journal de l'École Royale Polytechnique **18**, 123–140 (1845), in French, available at https://gallica.bnf.fr/ark:/12148/bpt6k4336867/f126, last accessed 6 June 2024.

[29] Pafnuty Chebyshev, *Mémoire sur les nombres premiers.* Journal de mathématiques pures et appliquées, Série 1 , 366–390 (1852), in French, available at http://www.numdam.org/item/JMPA_1852_1_17__366_0/, last accessed 6 June 2024.

[30] N. J. A. Sloane and The OEIS Foundation Inc., "The online encyclopedia of integer sequences". Retrieved from `http://oeis.org/` on February 27, 2025.

[31] They are all products of 2 and another odd prime, so the tensor products of the Wootters–Fields constructions will only guarantee 3 MUBs in $d = 6, 10, 14,$ and 22.
